# Macrophage M2 Co-expression Factors Correlate With the Immune Microenvironment and Predict Outcome of Renal Clear Cell Carcinoma

**DOI:** 10.3389/fgene.2021.615655

**Published:** 2021-02-22

**Authors:** Yutao Wang, Kexin Yan, Jiaxing Lin, Jun Li, Jianbin Bi

**Affiliations:** ^1^Department of Urology, The First Hospital of China Medical University, China Medical University, Shenyang, China; ^2^Department of Dermatology, The First Hospital of China Medical University, China Medical University, Shenyang, China

**Keywords:** M2 macrophage, weighted gene co-expression network analysis, tumor-associated macrophage, immune phenotype, risk model

## Abstract

**Purpose:** In the tumor microenvironment, the functional differences among various tumor-associated macrophages (TAM) are not completely clear. Tumor-associated macrophages are thought to promote the progression of cancer. This article focuses on exploring M2 macrophage-related factors and behaviors of renal clear cell carcinoma.

**Method:** We obtained renal clear cell carcinoma data from TCGA-KIRC-FPKM, GSE8050, GSE12606, GSE14762, and GSE3689. We used the “Cibersort” algorithm to calculate type M2 macrophage proportions among 22 types of immune cells. M2 macrophage-related co-expression module genes were selected using weighted gene co-expression network analysis (WGCNA). A renal clear cell carcinoma prognosis risk score was built based on M2 macrophage-related factors. The ROC curve and Kaplan–Meier analysis were performed to evacuate the risk score in various subgroups. The Pearson test was used to calculate correlations among M2 macrophage-related genes, clinical phenotype, immune phenotype, and tumor mutation burden (TMB). We measured differences in co-expression of genes at the protein level in clear renal cell carcinoma tissues.

**Results:** There were six M2 macrophage co-expressed genes (F13A1, FUCA1, SDCBP, VSIG4, HLA-E, TAP2) related to infiltration of M2 macrophages; these were enriched in neutrophil activation and involved in immune responses, antigen processing, and presentation of exogenous peptide antigen via MHC class I. M2-related factor frequencies were robust biomarkers for predicting the renal clear cell carcinoma patient clinical phenotype and immune microenvironment. The Cox regression model, built based on M2 macrophage-related factors, showed a close prognostic correlation (AUC = 0.78). The M2 macrophage-related prognosis model also performed well in various subgroups. Using western blotting, we found that VSIG4 protein expression levels were higher in clear renal cell carcinoma tissues than in normal tissues.

**Conclusion:** These co-expressed genes were most related to the M2 macrophage phenotype. They correlated with the immune microenvironment and predicted outcomes of renal clear cell carcinoma. These co-expressed genes and the biological processes associated with them might provide the basis for new strategies to intervene via chemotaxis of M2 macrophages.

## Introduction

Renal clear cell carcinoma (RCC) accounts for 80–90% of all renal cell carcinomas; clear cell carcinoma is not sensitive to chemotherapy and radiotherapy (Hsieh et al., 2017). For this reason, radical surgery has become the main treatment method. In clinical practice, although radical nephrectomy can benefit mostly patients, 30% of patients experience distant metastases after surgery (Motzer et al., 2013). Although we have adopted various treatment strategies for these patients with poor status, the long-term outcomes are not ideal (Linehan and Ricketts, 2019). With the development of immunotherapy in recent years, there have been studies showing that immunotherapy can benefit patients with renal clear cell cancer (Chowdhury and Drake, 2020; Díaz-Montero et al., 2020; Wang C. et al., 2020).

Renal clear cell carcinoma is characterized by many new tumor antigen peptides and high mutation burden; it is relatively sensitive to immunotherapies such as targeting PD1 and PD-L1 (Wang C. et al., 2020). Immune regulation plays a crucial role in the renal clear cell carcinoma microenvironment. This process includes immune checkpoints [mainly programmed cell death 1 (PD-1) and programmed cell death 1 ligand 1 (PD-L1)], as well as regulatory T cells, the original source of suppressor cell tumor-associated macrophages, and type 2 innate and adaptive lymphocytes (Xu W. et al., 2020). Macrophages in the primary or secondary tumor tissues are called tumor-associated macrophages (TAMs); these are the largest number of macrophages in the tumor stroma (Herberman et al., 1979). In recent years, clinical and experimental evidence has shown that macrophages promote the progression and metastasis of solid tumors, and this is somewhat different from our previous understanding (Pollard, 2004; Karnevi et al., 2014). Tumor-associated macrophages are divided into two types, M1 and M2 (Herberman et al., 1979; DeNardo and Ruffell, 2019). The biological effects of the two types are exact opposites. As tumors progress, increasing numbers of M2 macrophages appear, resulting in a weaker antigen presentation effect. For this reason, targeting macrophages has become a new therapeutic strategy (DeNardo and Ruffell, 2019). M1 type macrophages, namely, classically activated macrophages, highly express IL-12 and IL-23 that enhance antitumor effects (Lawrence and Natoli, 2011). By contrast, M2 type macrophages, namely, alternatively activated macrophages, promote tumor formation and development (Cervantes-Villagrana et al., 2020). The mechanism of this polarization of macrophages is not clear. This article focuses on exploring the M2 macrophage-related genes in renal clear cell cancer, and constructing co-expression networks of M2 macrophages using the WGCNA method. The results of this paper revealed the underlying interaction mechanisms of M2 macrophage co-expressing factors and explained the role of M2 macrophages in the immune microenvironment from the perspective of bioinformatics.

## Methods

### Macrophage M2, Tumor Purity, and Tumor Mutation Burden Evaluation

We downloaded The Cancer Genome Atlas TCGA—KIRC FPKM data (http://cancergenome.nih.gov/) containing 539 renal clear cell cancer tissue samples and 72 normal tissues. GSE8050 (Weinzierl et al., 2008), GSE12606 (Stickel et al., 2009), GSE14762 (Wang et al., 2009), and GSE36895 (Peña-Llopis et al., 2012) were also downloaded from the GEO (http://www.ncbi.nlm.nih.gov/geo/) database. The Robust Multi-Array Average (RMA) algorithm of the “sva” (Leek et al., 2012) package was used to remove batch effects among the four GEO cohorts. The TCGA cohort was used to select M2-related genes. Four GEO cohorts were combined using “sva” packages and to verify the results. The Cell type Identification By Estimating Relative Subsets Of RNA Transcripts (CIBERSORT) is a deconvolution algorithm based on a gene expression profile that characterizes the cell composition of complex tissues, quantifies immune cells, and accurately estimates the immune components of tumor samples. It expands the potential of the genomic database, showing the pattern of Renal Clear Cell Carcinoma with comprehensive immune cells. We calculated macrophage M2 cell proportions based on the LM22 matrix using the CIBERSORT (Chen et al., 2018) algorithm, Cibersort was used as an obvious method to evaluate the significance of infiltration of immune cells in the samples. The assessment results of some samples were not statistically significant, and we used *P* < 0.05 to screen the samples. The Estimation of Stromal and Immune cells in Malignant Tumor tissues using Expression data (ESTIMATE) is a method that infers the fraction of stromal and immune cells using gene expression signatures (Yoshihara et al., 2013). Using the ESTIMATE package, we calculated tumor purity in each renal clear cell cancer sample. TMB (tumor mutation burden) per megabyte is calculated by dividing the total number of mutations by the size of the target coding region (Li et al., 2020; Yang et al., 2020).

### Macrophage M2 Co-expression Network Conduction

Weighted gene co-expression network analysis (WGCNA) is a system biology approach that converts co-expression correlations into connection weights or topology overlap values (Langfelder and Horvath, 2008). We used this method to determine proportions of co-expressed genes in the M2 macrophage. The expression patterns are similar for genes with the same biological process and biological function (Jiang et al., 2017). We built a scale-free topology network, set the soft threshold at 5, *R* square = 0.89, and set the number of genes in the minimum module at 30. The M2 macrophage cell proportion was considered for phenotype files in WGCNA. In this manner, a cluster of M2 macrophage cell proportion-related genes with similar function were identified in the same module. The factors with M2 macrophage correlation >0.4 in the most relevant modules were determined.

### M2 Macrophage-Related Module Analysis

The genes were selected using |correlation coefficient| > 0.4. The Database for Annotation, Visualization and Integrated Discovery (DAVID, v6.8) is an open-source database that performs function enrichment (Huang et al., 2007). We used the Kyoto Encyclopedia of Genes and Genomes (KEGG) (https://www.genome.jp/kegg/) (Kanehisa et al., 2017) and Gene Ontology (GO) (http://geneontology.org/) analysis (Ashburner et al., 2000) to identify the biological function in each co-expression module. In this way, we identified the biological processes associated with M2-type macrophage proportion.

### M2 Macrophage-Related Genes Analysis

To verify the correlation between these factors and the clinical phenotype, we measured the overall survival from clear cell carcinoma as the prognostic indicator. Survival analysis was performed to evaluate the prognostic value of these co-expressed factors in M2 macrophages. Subsequently, a Cox regression hazard model was built based on the M2 macrophage-related genes. Next, we generated a model validation of clinical subgroups, which was based on age, gender, tumor metastasis, tumor stage, tumor purity, and degree of tumor mutation burden. In different subgroups, we evaluated the predictive abilities of M2 macrophage-related prognostic models. Finally, we calculated tumor purity in TCGA samples and explored the correlations between macrophage-related factors and tumor purity.

### HPA

To verify the protein expression levels of candidate genes in melanoma and normal tissues, the human protein atlas (HPA, https://www.proteinatlas.org/) database was used to demonstrate differences in co-expressed genes at the protein level (Uhlén et al., 2015).

### Western Blotting

Thirty clear renal cell carcinoma tissue samples were obtained from patients who underwent Nephrectomy at the First Affiliated Hospital of China Medical University. This study was authorized by the Ethics Committee of the First Affiliated Hospital of China Medical University. All patients signed informed consent. Protein exaction and western blotting were conducted as described previously (Pripp, 2018). An antibody against VSIG4 was purchased from Sigma-Aldrich.

### Statistical Methods

Pearson correlation coefficients measure the strength of the linear relationship between two variables. The correlation coefficients are −1 to +1, respectively, indicating negative correlation and positive correlation, respectively, while 0 indicates no correlation (Wang Y. et al., 2020). The key factors in the model score, tumor purity, tumor mutation burden, M2 macrophages, and CD8^+^ T lymphocytes were assessed using this test.

## Results

### M2 Macrophages, Tumor Purity, and Tumor Mutation Burden

**Figure 1:**
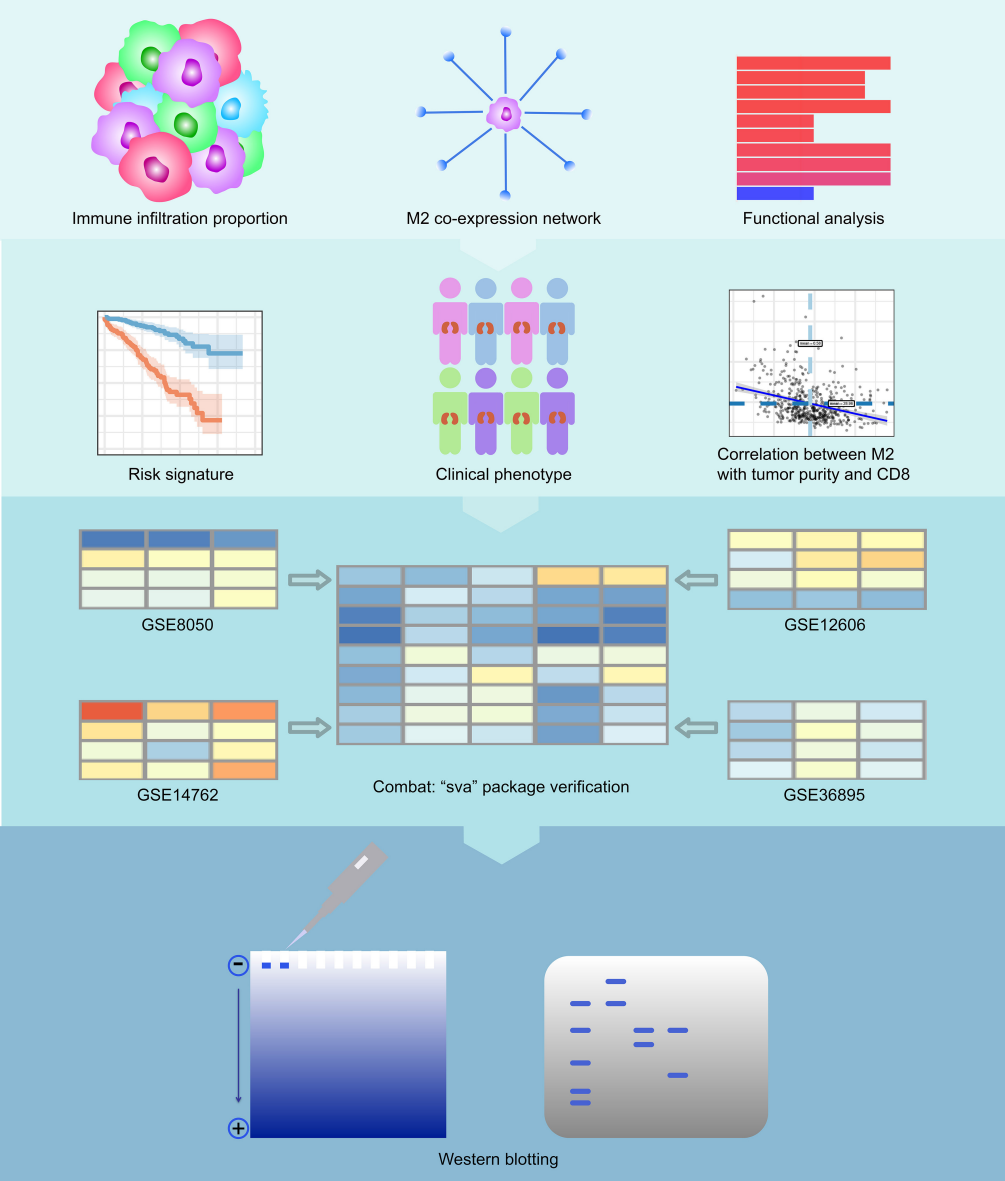
Flowchart of the experimental design. We first calculated immune infiltration to determine the content of M2 macrophages in the immune microenvironment of RCC. Then, we constructed a co-expression network related to M2 macrophages of RCC and analyzed the enriched pathways in this network. We then calculated the survival analysis of these co-expressed genes. We constructed a COX regression prognostic model associated with the co-expression genes of M2 macrophages in RCC and performed a subgroup analysis of this model. We analyzed the relationship between key genes in the model and tumor purity and CD8^+^ T cells. Finally, we also verified the feasibility of the model with 4 GEO datasets and conducted western blotting experiments on VSIG4.

The results of our methodology are explained in Figure 1.

We summed up the following clinical data composed by M2 macrophages, tumor mutation burden, and clinical following survival data. M2, and M1, and M2/M1 macrophages were inputted as phenotype files to WGCNA. The detailed information is displayed in Supplementary Table 1.

### M2 Macrophages Co-expression Network Conduction

**Figure 2:**
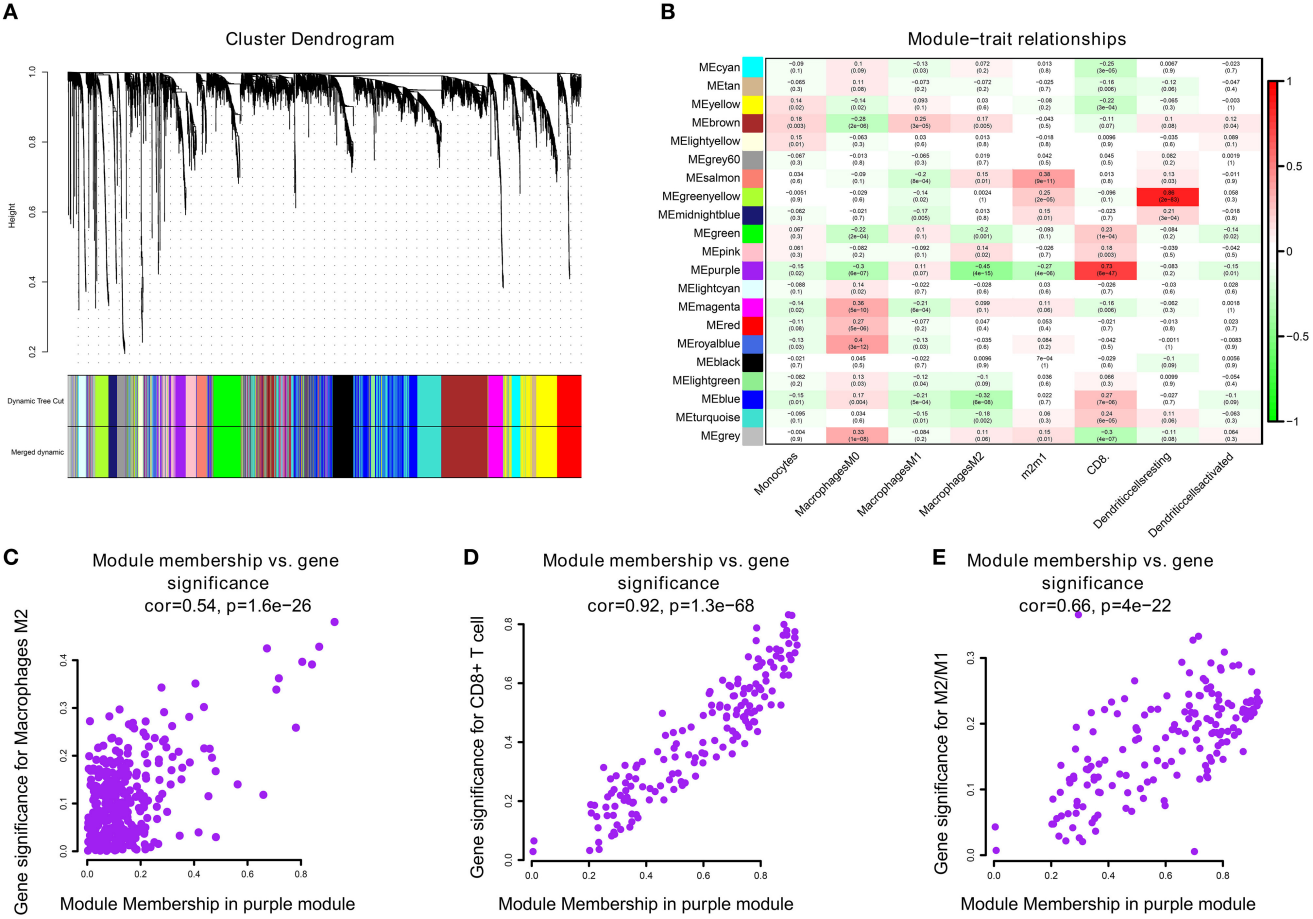
**(A)** A hierarchical clustering tree was built using the dynamic hybrid cutting method, where each leaf on the tree represents a gene, and each branch represents a co-expression module; 21 co-expression models were generated. **(B)** The correlation coefficients between each phenotype and co-expression module of TCGA. The purple module had the strongest correlation with M2 macrophage cell proportions in the TCGA–KIRC cohort (Cor = −0.45; *P* = 4e^−15^) and had the strongest correlation with CD8^+^ T cell proportions in the TCGA–KIRC cohort (Cor = 0.73; *P* = 6e^−47^). **(C)** The relationship between the purple module membership degree and the gene significance of M2 macrophages (cor = 0.54; *P* = 1.6e−26). **(D)** The relationship between the purple module membership degree and the gene significance of CD8+ T Cells (cor = 0.92; *P* = 1.3e−68). **(E)** The relationship between the purple module membership degree and the gene significance of M2/M1 ratio (cor = 0.66; *P* = 4e−22).

We performed WGCNA analysis with TCGA–KIRC. A hierarchical clustering tree was built using the dynamic hybrid cutting method, where each leaf on the tree represents a gene, and each branch represents a co-expression module; 21 co-expression models were generated (Figure 2A). The correlation coefficients between each phenotype and co-expression module of TCGA are shown in Figure 2B. The results showed that the purple module had the strongest negatively correlation with M2 macrophage cell proportion in the TCGA–KIRC cohort (Cor = −0.45; *P* = 4e^−15^) and had the strongest correlation with CD8^+^ T cell proportion in the TCGA–KIRC cohort (Cor = 0.73; *P* = 6e^−47^) (Figure 2B). Based on these findings, we have supplemented the scatter plots of the correlation between the factors in the purple module (Figures 2C–E). The horizontal axis is the correlation between the gene and the module, which is used to measure the relationship between the gene and the co-expression module, and the vertical axis is the correlation between the gene and the macrophage. By drawing the scatter diagram above, we screened out genes that are related to both M2 macrophages and the co-expression module.

### M2 Related Genes Function Analysis

**Figure 3:**
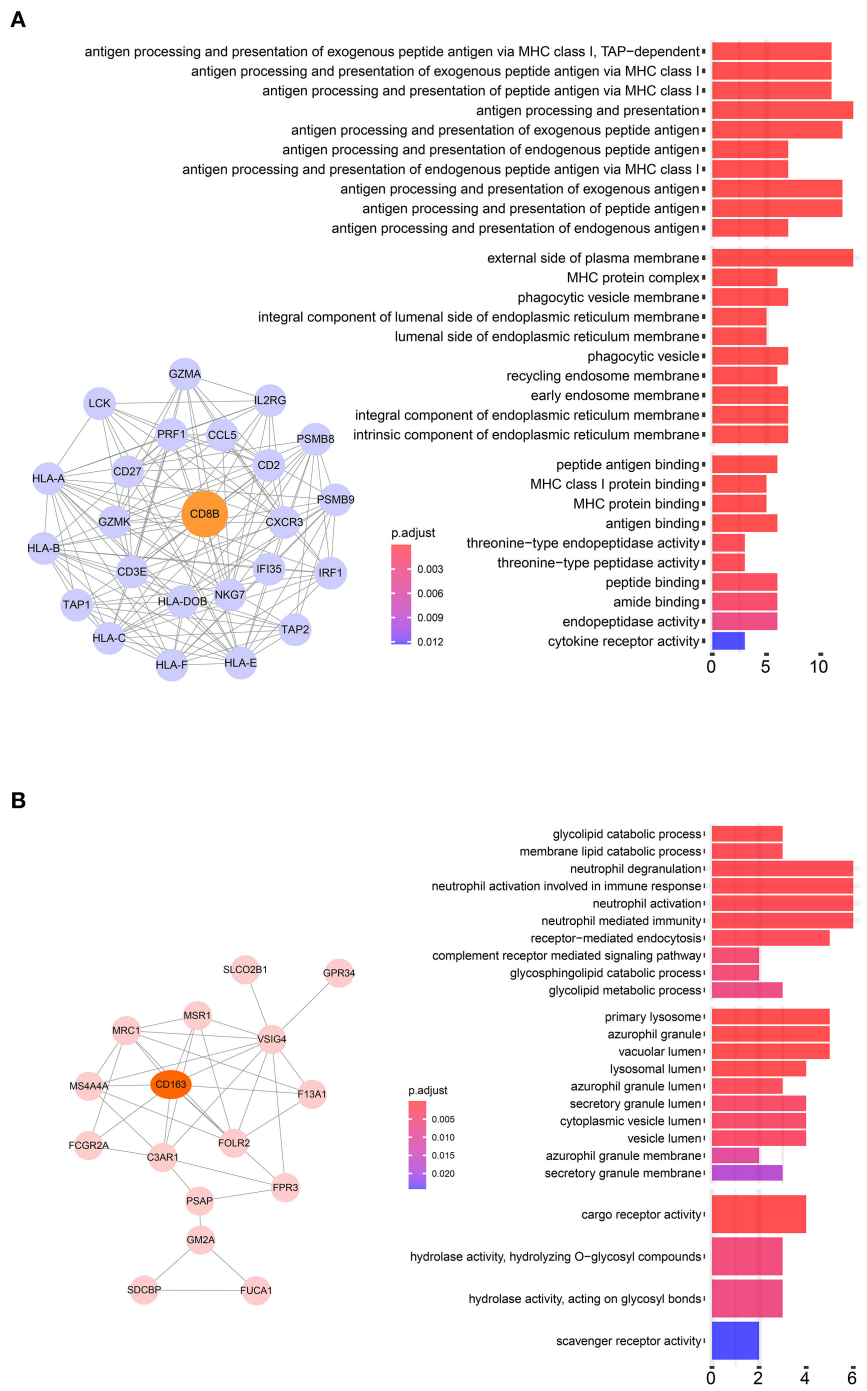
**(A)** Pathway analysis of 24 negatively correlated co-expressed genes in M2 macrophages in the purple module. These genes were most significantly enriched in the antigen processing and presentation of exogenous peptide antigen via MHC class I, which suggested a declining effect on tumor antigen peptide process. **(B)** Pathway analysis of 16 negatively correlated co-expressed genes in M2 macrophages in the brown module. These genes were most significantly enriched in neutrophil activation involved in immune responses.

Twenty-four M2 macrophage negatively co-expressing genes were identified with coefficient <-0.4 in the TCGA–KIRC purple module. The gene significance for M2 macrophage-related genes in the purple module is shown in Table 1. Top 20 M2 macrophage cell proportion positively co-expressing genes were identified in the TCGA–KIRC pink module. The 24 M2 macrophage negatively co-expressing genes were most significantly enriched in the antigen processing and presentation of exogenous peptide antigen via MHC class I, which suggested a declining effect on the tumor antigen peptide process (Figure 3A). The 20 M2 macrophage negatively co-expressing genes were most significantly enriched in neutrophil activation involved in immune responses (Figure 3B).

**Table 1:** The Module and gene significance for M2 macrophage-related genes in the purple module.

**ID**	**moduleColor**	**GS.MacrophagesM2**	**p.GS.M2**	**GS.CD8.T**	**p.GS.CD8**.
CD27	purple	−0.497	1.32E-18	0.774	2.91E-56
PSMB9	purple	−0.493	2.94E-18	0.715	1.65E-44
CTSW	purple	−0.488	7.07E-18	0.787	2.57E-59
CD3E	purple	−0.483	1.57E-17	0.734	6.70E-48
CST7	purple	−0.482	1.77E-17	0.799	2.39E-62
CD3D	purple	−0.480	2.90E-17	0.755	5.32E-52
SIT1	purple	−0.479	3.05E-17	0.753	1.02E-51
HLA-F	purple	−0.476	5.88E-17	0.689	3.94E-40
IL2RG	purple	−0.475	6.28E-17	0.649	2.22E-34
GZMA	purple	−0.468	2.22E-16	0.7789	2.89E-57
NKG7	purple	−0.468	2.22E-16	0.762	1.28E-53
CD8B	purple	−0.467	2.28E-16	0.832	6.65E-72
PRF1	purple	−0.466	2.80E-16	0.744	9.06E-50
CD8A	purple	−0.466	2.93E-16	0.830	3.40E-71
LCK	purple	−0.465	4.62E-16	0.694	2.54E-42
APOBEC3G	purple	−0.461	6.88E-16	0.715	2.18E-44
HLA-B	purple	−0.459	9.61E-16	0.682	4.66E-39
CXCR3	purple	−0.458	1.03E-15	0.684	2.29E-39
IRF1	purple	−0.457	1.30E-15	0.671	2.24E-37
CD2	purple	−0.449	4.11E-15	0.729	6.85E-47
DUSP2	purple	−0.449	4.43E-15	0.763	1.13E-53
CCL5	purple	−0.447	5.76E-15	0.588	4.94E-27
HLA-E	purple	−0.423	1.08E-14	0.688	1.58E-35
PSME2	purple	−0.409	1.98E-14	0.525	1.11E-43

*GS, Gene significance*.

### M2 Related Genes Prognosis Analysis

**Figure 4:**
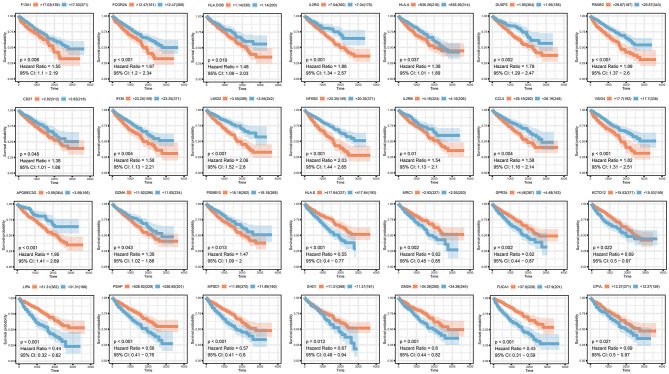
Survival analysis of selected co-expressed genes in purple and pink modules.

To analyze their influence on overall survival, we performed survival analysis. F13A1, FCGR2A, HLA.DOB, ILR2GHLA, DUSP2, PSME2, CD27, IFI35, LIMD2, NFKB2, IL2RB, CCL5, VSIG4, APOBEC3G, GZMA, and PSMB10 were prognosis risk factors for clear renal cell carcinoma. HLA-E, MRC1, GPR34, KCTD12, LIPA, PSAP, MFSD1, EHD1, FUCA1, and CPVL were prognosis-protective factors for clear renal cell carcinoma (Figure 4).

### M2 Macrophage-Related Prognosis Signature

**Figure 5:**
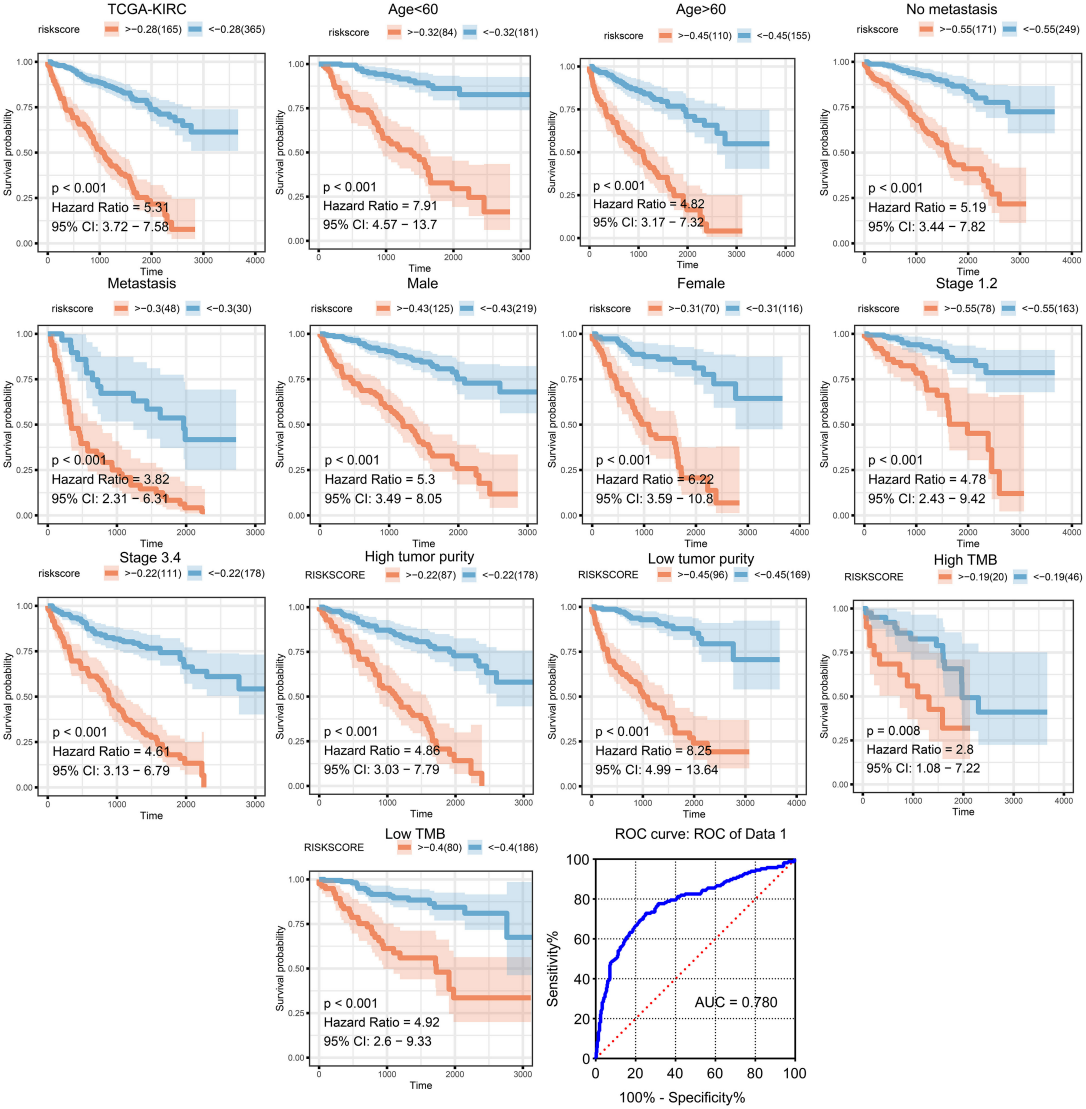
Validation of the prognostic model in clinical subgroups. The patients in high-risk groups for renal clear cell cancer (TCGA: *P* < 0.001; HR = 5.31) showed survival risk against low expression groups, with the area under curve (AUC) = 0.780. The risk score was evaluated in clinical subgroups, including age, gender, stage, metastasis, tumor purity, and tumor mutation burden. P-values of all subgroups validations were <0.05, indicating that this model has good predictive ability.

We then generated a multi-Cox regression risk score model based on M2 macrophage-related genes (Tables 1, 2). Risk score = 0.025 ^*^ F13A1 – 0.008 ^*^ FUCA1 + 0.034 ^*^ FCGR2A – 0.016 ^*^ KCTD12 – 0.08 ^*^ MFSD1 – 0.003 ^*^ HLA-E + 0.012 ^*^ SDCBP – 0.071 ^*^ MRC1 – 0.086 ^*^ LCK + 0.02 ^*^ PSME2 + 0.016 ^*^ VSIG4 + 0.215 ^*^ TAP2. Detailed information of the prognosis model is displayed in Supplementary Table 2. The patients in high-risk groups for renal clear cell cancer (TCGA: *P* < 0.001; HR = 5.31) (Figure 5) showed survival risk vs. low expression groups, with the area under the curve (AUC) = 0.780 (Figure 5). The risk score was evaluated in various subgroups, including age, gender, stage, metastasis, tumor purity, and tumor mutation burden. The results were significant in these subgroups (Figure 5).

**Table 2:** The Module and gene significance for M2 macrophage-related genes in the pink module.

**ID**	**moduleColor**	**GS.MacrophagesM2**	**p.GS.M2**
GPR34	pink	0.467	2.31E-16
MS4A4A	pink	0.452	2.93E-15
MFSD1	pink	0.446	6.88E-15
FUCA1	pink	0.435	3.55E-14
CD163	pink	0.428	1.07E-13
FOLR2	pink	0.427	1.13E-13
LIPA	pink	0.424	1.78E-13
SLCO2B1	pink	0.418	4.43E-13
PSAP	pink	0.415	6.44E-13
SDCBP	pink	0.404	3.17E-12
C3AR1	pink	0.395	9.95E-12
F13A1	pink	0.391	1.60E-11
KCTD12	pink	0.386	3.14E-11
MSR1	pink	0.384	4.12E-11
CPVL	pink	0.365	4.15E-10
FCGR2A	pink	0.362	5.48E-10
FPR3	pink	0.358	9.40E-10
GM2A	pink	0.353	1.68E-09
VSIG4	pink	0.347	3.12E-09
MRC1	pink	0.337	9.28E-09

*GS, Gene significance*.

### Immune Environment Correlation

**Figure 6:**
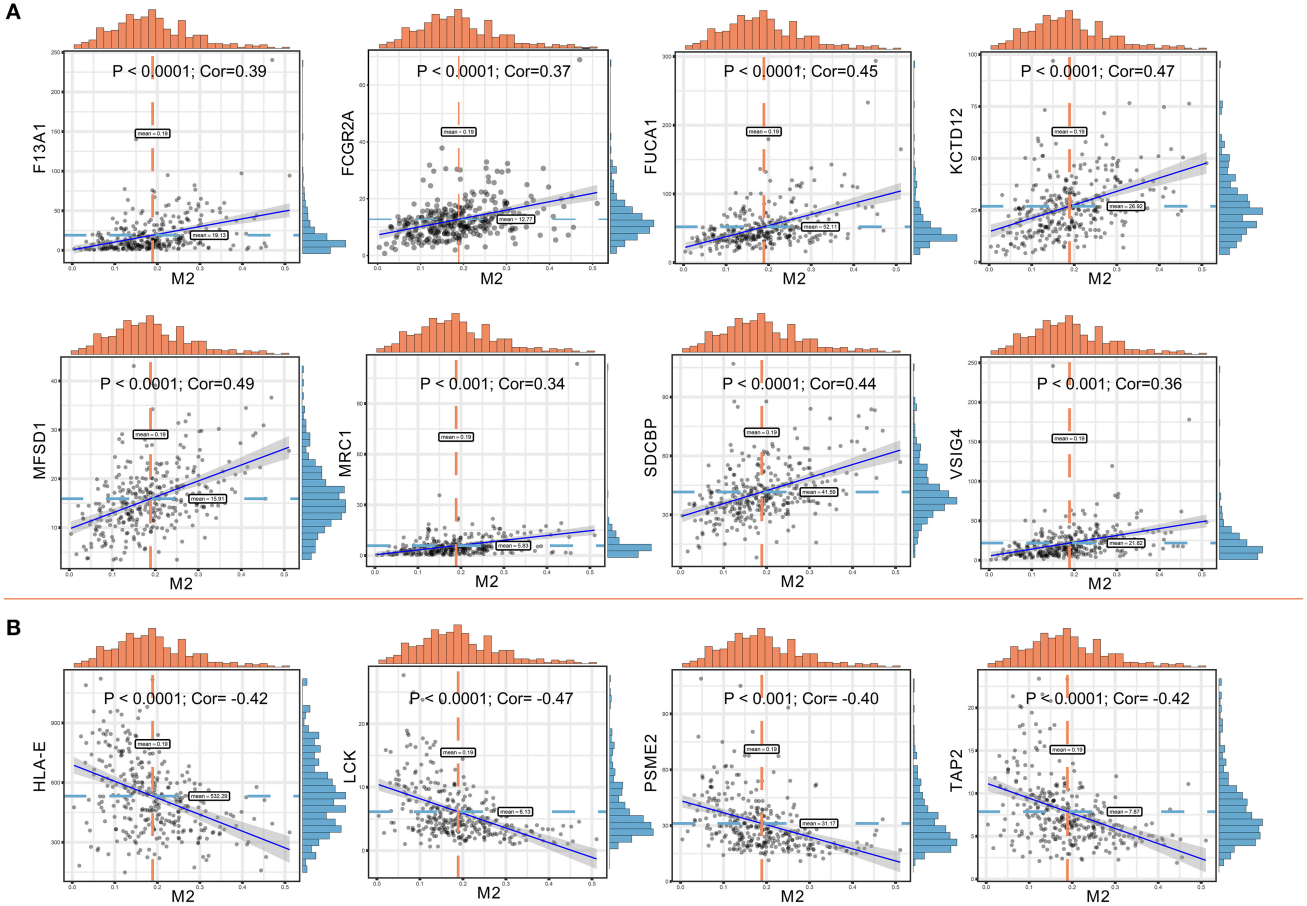
**(A)** Co-expressed genes with a significant positive correlation with M2 macrophages. The correlation coefficients are as follows: F13A1 – M2: Cor = 0.39; FCGR2A – M2: Cor = 0.37; FUCA1 – M2: Cor = 0.45; KCTD12 – M2: Cor = 0.47; MFSD1 – M2: Cor = 0.49; MRC1 – M2: Cor = 0.34; SDCBP – M2: Cor = 0.44; VSIG4 – M2: Cor = 0.36. **(B)** Co-expressed genes with a significant negative correlation with M2 macrophages. The correlation coefficients are as follows: HLA-E – M2: Cor = −0.42; LCK – M2: Cor = −0.47; PSME2 – M2: Cor = −0.40; TAP2 – M2: Cor = −0.42.

**Figure 7:**
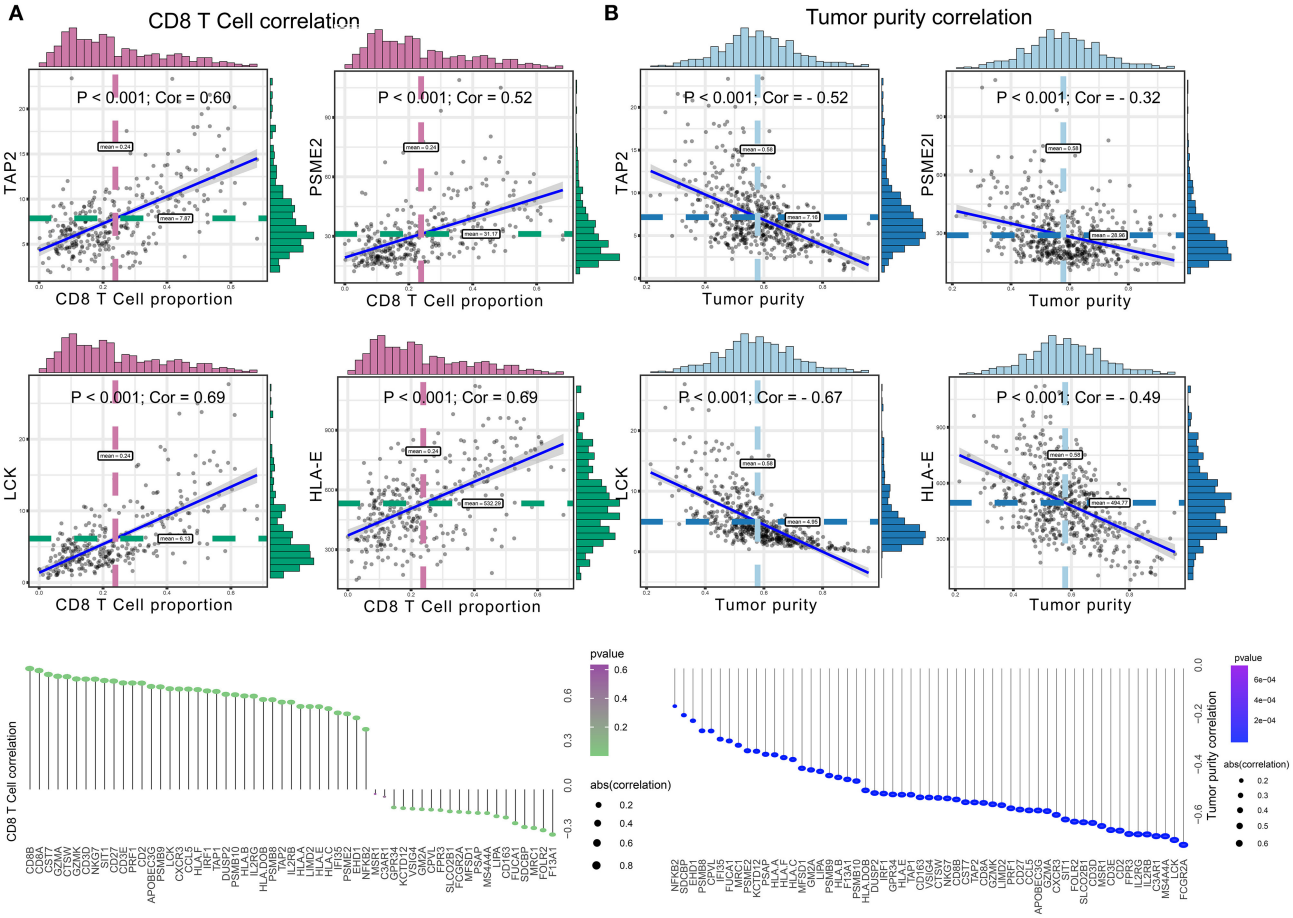
**(A)** The correlation between co-expressed gene of M2 macrophage and CD8^+^ T cell, with significantly positive relations as TAP2 (*P* < 0.001; Cor = 0.60), PSME2 (*P* < 0.001; Cor = 0.52), HLA – E (*P* < 0.001; Cor = 0.69), and LCK (*P* < 0.001; Cor = 0.69). **(B)** The correlation between co-expressed genes of M2 macrophage and tumor purity, with significantly negative relations as TAP2 (*P* < 0.001; Cor = −0.52), PSME2 (*P* < 0.001; Cor = −0.32), LCK (*P* < 0.001; Cor = −0.67), and HLA-E (*P* < 0.001; Cor = −0.49).

Significant associations between M2 frequency and the genes involved in the risk signature are indicated in Figure 6, and the highest correlation of MFSD1 was 0.49 (Figure 6A); the correlation of LCK was the lowest at −0.47 (Figure 6B). TAP2, PSME2, HLA-E, and LCK were negatively related to M2 macrophage proportions. We then analyzed the correlations with CD8^+^ T cell and tumor mutation burden of these four genes. TAP2 (*P* < 0.001; Cor = 0.60), PSME2 (*P* < 0.001; Cor = 0.52), HLA - E (*P* < 0.001; Cor = 0.69), and LCK (*P* < 0.001; Cor = 0.69) (Figure 7A) positively related to CD8^+^ T cell and negatively correlated with tumor purity (Figure 7B). This result suggested that M2 macrophages were negatively related to antigen processing.

### HPA

**Figure 8:**
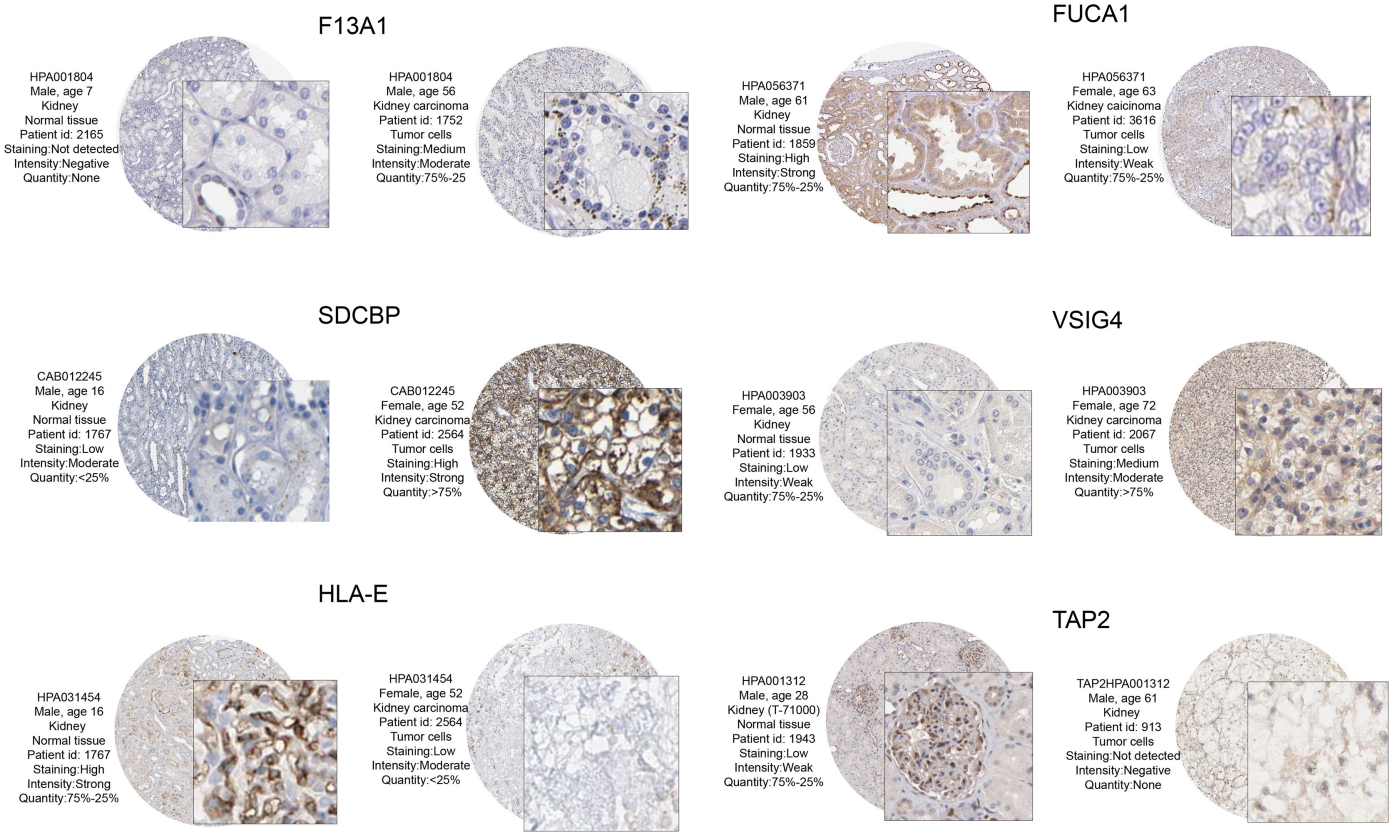
From the HPA database to verify protein expression-level differences of these candidate genes. Of these, F13A1, SDCBP, and VSIG4, and corresponding immunohistochemical samples, the degree of renal clear cell carcinoma tissue staining is higher than in normal kidney tissue. In FUCA1, HLA – E, and TAP2, and corresponding immunohistochemical samples, the degree renal clear cell carcinoma tissue staining is lower than in normal kidney tissue. These M2 macrophage gene protein levels at the transcription level were similar to those of the prognostic analysis.

The prognostic value and immune phenotype correlation were determined for these M2 macrophage-related genes. We compared the various expression levels of these genes between normal and tumor tissues. HPA001804 is an antibody against F13A1, which showed higher intensity in tumor tissue than in normal tissue. HPA056371 is an antibody against FUCA1, which showed higher intensity in the normal tissue than in tumor tissue. CAB012245 is an antibody against SDCBP, which showed a higher intensity in tumor tissue than in normal tissue. HPA003903 is an antibody against VSIG4, which showed higher intensity in tumor tissue than in normal tissue. HPA031454 is an antibody against HLA-E, which showed lower intensity in tumor tissue than in normal tissue. HPA001312 is an antibody against TAP2, which showed lower intensity in tumor tissue than in normal tissue. The protein levels of these M2 macrophage genes were similar to the results of prognosis analysis at the transcription level (Figure 8). Subsequently, the M2 correlations for VSIG4, FUCA1, F13A1, SDCBP, HLA-E, and TAP2 were verified in the four GEO datasets (Supplementary Figure 1).

### VSIG4

**Figure 9:**
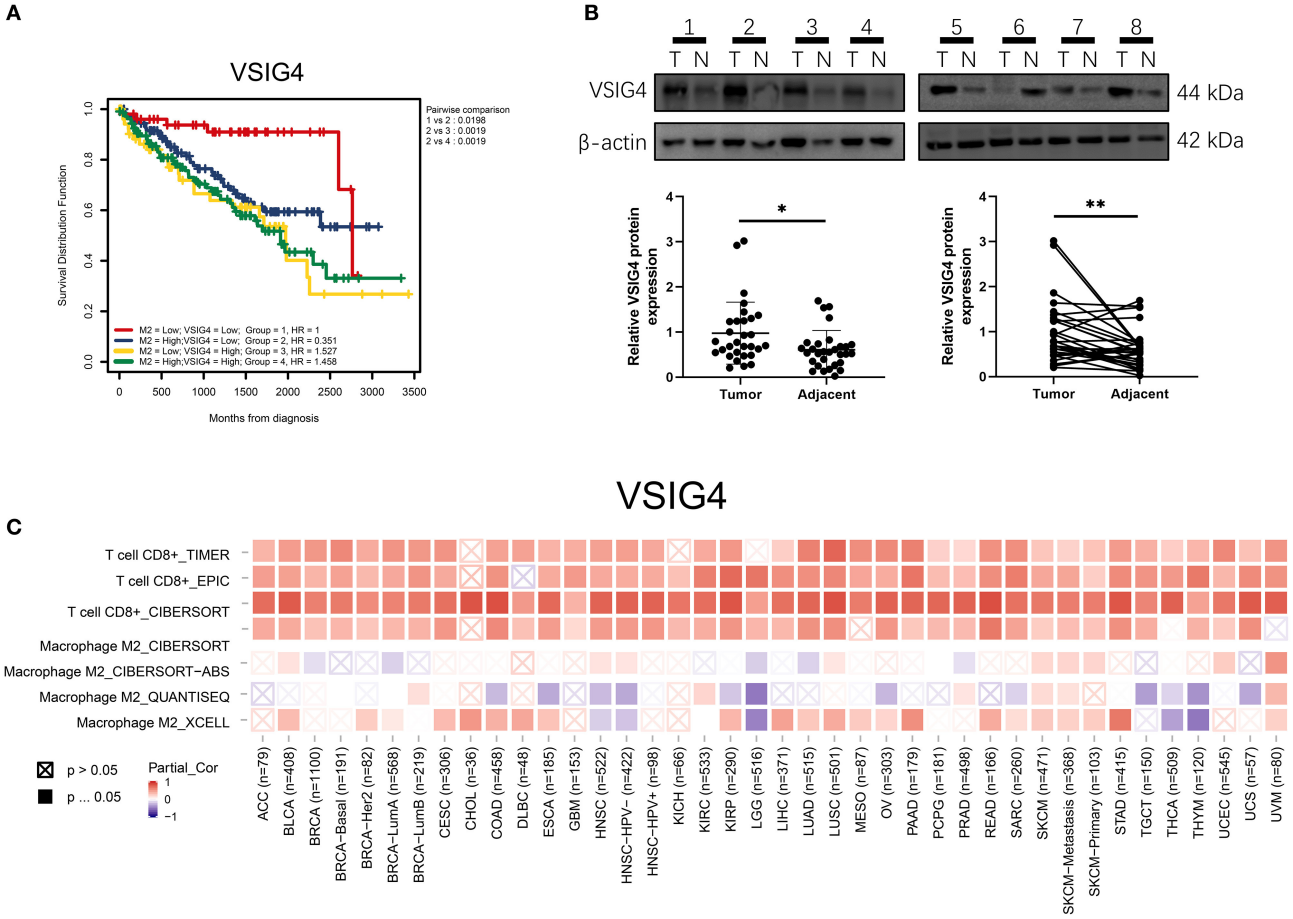
**(A)** Combining high VSIG4 and high M2 macrophage showed more survival risk than the other group. **(B)** The VSIG4 protein expression levels were higher in clear renal cell carcinoma tissues than in normal tissues according to western blotting. **(C)** Pan-cancer analysis of VSIG4 in TCGA.

VSIG4 is thought to positively correlate with M2 macrophages; therefore, we conducted a combined analysis of VSIG4 and M2-type macrophages. Combining VSIG4 elevated the predictive accuracy of M2 macrophages even more than either of them alone; the hazard of the “high VSIG4 expression + high M2 macrophage” group showed more survival risk than the other group (Kaplan–Meier analysis, low VSIG4 expression + low M2 macrophage; HR = 1.458; Figure 9A). Subsequently, we compared VSIG4 protein expression levels between normal renal tissues and clear renal cell carcinoma and found that VSIG4 protein expression levels in tumors were higher than the normal tissues (Figure 9B). Then, various tumor infiltration deconvolution methods were applied; we found that VSIG4 was one of the most commonly associated M2 macrophage biomarkers (Figure 9C).

## Discussion

In the tumor microenvironment, the chemotactic effects of the functional differences between the types of tumor-associated macrophages are not completely clear. The biological cytological role of M2/M1 macrophages in tumor tissues still needs to be explored. The present study is based on a bioinformatics algorithm to determine some of the M2 macrophage co-expression networks. Through the analysis of various modules, we tried to explain the biological function of co-expressed genes with M2 macrophages and related pathway changes from the perspective of bioinformatics. Our data processing and analysis processes are shown in the flowchart (Figure 1).

F13A1, FCUA1, HLA-E, VSIG4, SDCBP, and TAP2 were the most common co-expressed genes in M2 macrophages. In terms of function enrichment, the 24 negatively co-expressed genes in M2 macrophages were most significantly enriched in antigen processing and presentation of exogenous peptide antigen via MHC class I. The 16 negatively co-expressing genes in M2 macrophages were most significantly enriched in neutrophil activation involved in immune response. M1 macrophages tend to adopt a Th1 response gene expression pattern and can secrete various cytokines that present MHC II and B7 molecules so as to present antigen efficiently (Herberman et al., 1979). This mechanism resists pathogen invasion, monitors tumor pathological changes, and generates Th1 immune responses in macrophages. By contrast, M2 macrophages have poor tumor antigen processing ability.

F13A1 encodes the coagulation factor XIII A subunit which has a catalytic function. In a human stem cell study, mRNA transcription expressed by F13A1 increased as myeloid progenitors differentiated into macrophages and erythroblasts (De Paoli et al., 2015). The protein encoded by FCUA1 is a lysosomal enzyme involved in the degradation of fucose-containing glycoproteins and glycolipids. Downregulation of FUCA1 enhances autophagy and inhibits macrophage infiltration so as to inhibit tumor growth (Xu L. et al., 2020). VSIG4 is a transmembrane receptor of the immunoglobulin superfamily that is specifically expressed in macrophages and mature dendritic cells. It is a newly discovered B7 family-related macrophage protein that inhibits T cell activation and has a potential role in cancer (Kim et al., 2016). VSIG4 negatively regulates macrophage activation by reprogramming mitochondrial pyruvate metabolism (Li et al., 2017). HLA-E belongs to the HLA class I heavy chain paralogs. This class I molecule is a heterodimer consisting of a heavy chain and a light chain (beta-2 microglobulin). The heavy chain is anchored in the membrane. HLA-E binds a restricted subset of peptides derived from the leader peptides of other class I molecules. HlA-E is a non-classical HLA-I molecule that is best known for its role in protecting natural killer cells. Camilli et al. found that HLA-E was significantly increased during the differentiation of monocytes and macrophages (Camilli et al., 2016). The expression of HLA-E is related to the poor clinical results of anti-PD-1 immunotherapy. From the surface of M2 tumor-associated macrophages (TAMs), HLA-E antigen binds to the receptor CD94/NKG2A, which inhibits the expression of NK cell subpopulations and activated cytotoxic T lymphocytes, protecting cells from being destroyed (Marchesi et al., 2013). Epithelial-derived cancer cells, tumor macrophages, and CD141^+^ traditional dendritic cells promote the enrichment of HLA-E in carcinomas. CD8^+^ tumor-infiltrating T lymphocytes with high PD-1 content are prevented from surviving in the tumor microenvironment by the interaction of enriched HLA-E and CD94/NKG2A inhibition (Abd Hamid et al., 2019).

This study has some limitations, including lack of cross-validation of multicenter data. There is also lack of experimental verification of M2 macrophage biomarkers in renal clear cell cancer. We found that using the co-expression method of network-building, we can explicitly identify biomarkers, demonstrating the correctness of the logic based on bioinformatics.

In conclusion, we found that F13A1, FCUA1, HLA-E, VSIG4, SDCBP, and TAP2 were biomarkers of M2-type macrophages using a co-expression network of infiltrated immune cells, and we proposed six candidate-related factors. The biomarkers and related processes of M2 macrophages in the tumor microenvironment were explained from the perspective of bioinformatics, providing a strategy to explore the polarization of macrophages.

## Data Availability Statement

Publicly available datasets were analyzed in this study. This data can be found here: The TCGA-BLCA dataset used in this study were obtained from TCGA database (https://cancergenome.nih.gov/). GEO datasets used in this study were obtained from GEO database (https://www.ncbi.nlm.nih.gov/geo/).

## Ethics Statement

The studies involving human participants were reviewed and approved by The First Affiliated Hospital of China Medical University. The patients/participants provided their written informed consent to participate in this study.

## Author Contributions

YW, KY, and JB designed the study. YW, KY, JLin, JLi, and JB analyzed and wrote the article. All authors read and agreed to the final version of the manuscript.

## Conflict of Interest

The authors declare that the research was conducted in the absence of any commercial or financial relationships that could be construed as a potential conflict of interest.

## Supplementary Material

Supplementary Figure 1 **(A)** The multi-dataset correction of GSE8050, GSE12606, GSE14762, and GSE36895 using the “sva” package. **(B)** The verification analysis of M2 related genes in the combined cohorts.

Supplementary Table 1 The detail information of tumor mutation, tumor purity, and M2 macrophage proportion.

Supplementary Table 2 The process data of multi-Cox regression risk score model.

## Abbreviations

TCGA: The Cancer Genome Atlas
GEO: Gene Expression Omnibus
BLCA: Bladder urothelial carcinoma
ROC: Receiver operating characteristic
AUC: Area under the curve
HR: Hazard ratio
TME: Tumor microenvironment.

## References

[B1] Abd Hamid M. Wang R. Z. Yao X. Fan P. Li X. Chang X. M.. (2019). Enriched HLA-E and CD94/NKG2A interaction limits antitumor CD8+ tumor-infiltrating T lymphocyte responses. Cancer Immunol. Res. 7, 1293–1306. 10.1158/2326-6066.CIR-18-0885 31213473

[B2] Ashburner M. Ball C. A. Blake J. A. Botstein D. Butler H. Cherry J. M.. (2000). Gene ontology: tool for the unification of biology. The Gene Ontology Consortium. Nat. Genet. 25, 25–29. 10.1038/75556 10802651 PMC3037419

[B3] Camilli G. Cassotta A. Battella S. Palmieri G. Santoni A. Paladini F.. (2016). Regulation and trafficking of the HLA-E molecules during monocyte-macrophage differentiation. J. Leukoc. Biol. 99, 121–130. 10.1189/jlb.1A0415-172R 26310830

[B4] Cervantes-Villagrana R. D. Albores-García D. Cervantes-Villagrana A. R. García-Acevez S. J. (2020). Tumor-induced neurogenesis and immune evasion as targets of innovative anti-cancer therapies. Signal Transduct. Target Ther. 5:99. 10.1038/s41392-020-0205-z 32555170 PMC7303203

[B5] Chen B. Khodadoust M. S. Liu C. L. Newman A. M. Alizadeh A. A. (2018). Profiling tumor infiltrating immune cells with CIBERSORT. Methods Mol. Biol. 1711, 243–259. 10.1007/978-1-4939-7493-1_12 29344893 PMC5895181

[B6] Chowdhury N. Drake C. G. (2020). Kidney cancer: an overview of current therapeutic approaches. Urol. Clin. North Am. 47, 419–431. 10.1016/j.ucl.2020.07.009 33008493

[B7] De Paoli F. Eeckhoute J. Copin C. Vanhoutte J. Duhem C. Derudas B.. (2015). The neuron-derived orphan receptor 1 (NOR1) is induced upon human alternative macrophage polarization and stimulates the expression of markers of the M2 phenotype. Atherosclerosis 241, 18–26. 10.1016/j.atherosclerosis.2015.04.798 25941992

[B8] DeNardo D. G. Ruffell B. (2019). Macrophages as regulators of tumour immunity and immunotherapy. Nat. Rev. Immunol. 19, 369–382. 10.1038/s41577-019-0127-6 30718830 PMC7339861

[B9] Díaz-Montero C. M. Rini B. I. Finke J. H. (2020). The immunology of renal cell carcinoma. Nat. Rev. Nephrol. 16, 721–735. 10.1038/s41581-020-0316-3 32733094

[B10] Herberman R. B. Holden H. T. Djeu J. Y. Jerrells T. R. Varesio L. Tagliabue A.. (1979). Macrophages as regulators of immune responses against tumors. Adv. Exp. Med. Biol. 121B, 361–379. 10.1007/978-1-4684-8914-9_35 232619

[B11] Hsieh J. J. Purdue M. P. Signoretti S. Swanton C. Albiges L. Schmidinger M.. (2017). Renal cell carcinoma. Nat. Rev. Dis. Primers 3:17009. 10.1038/nrdp.2017.9 28276433 PMC5936048

[B12] Huang D. W. Sherman B. T. Tan Q. Collins J. R. Alvord W. G. Roayaei J.. (2007). The DAVID Gene Functional Classification Tool: a novel biological module-centric algorithm to functionally analyze large gene lists. Genome Biol. 8:R183. 10.1186/gb-2007-8-9-r183 17784955 PMC2375021

[B13] Jiang J. Sun X. Wu W. Li L. Wu H. Zhang L.. (2017). Corrigendum: Construction and application of a co-expression network in Mycobacterium tuberculosis. Sci. Rep. 7:40563. 10.1038/srep40563 28079164 PMC5227706

[B14] Kanehisa M. Furumichi M. Tanabe M. Sato Y. Morishima K. (2017). KEGG: new perspectives on genomes, pathways, diseases and drugs. Nucleic Acids Res. 45, D353–D361. 10.1093/nar/gkw1092 27899662 PMC5210567

[B15] Karnevi E. Andersson R. Rosendahl A. H. (2014). Tumour-educated macrophages display a mixed polarisation and enhance pancreatic cancer cell invasion. Immunol. Cell Biol. 92, 543–552. 10.1038/icb.2014.22 24662521

[B16] Kim K. H. Choi B. K. Kim Y. H. Han C. Oh H. S. Lee D. G.. (2016). Extracellular stimulation of VSIG4/complement receptor Ig suppresses intracellular bacterial infection by inducing autophagy. Autophagy 12, 1647–1659. 10.1080/15548627.2016.1196314 27440002 PMC5082771

[B17] Langfelder P. Horvath S. (2008). WGCNA: an R package for weighted correlation network analysis. BMC Bioinformatics 9:559. 10.1186/1471-2105-9-559 19114008 PMC2631488

[B18] Lawrence T. Natoli G. (2011). Transcriptional regulation of macrophage polarization: enabling diversity with identity. Nat. Rev. Immunol. 11, 750–761. 10.1038/nri3088 22025054

[B19] Leek J. T. Johnson W. E. Parker H. S. Jaffe A. E. Storey J. D. (2012). The sva package for removing batch effects and other unwanted variation in high-throughput experiments. Bioinformatics 28, 882–883. 10.1093/bioinformatics/bts034 22257669 PMC3307112

[B20] Li J. Diao B. Guo S. Huang X. Yang C. Feng Z.. (2017). VSIG4 inhibits proinflammatory macrophage activation by reprogramming mitochondrial pyruvate metabolism. Nat. Commun. 8:1322. 10.1038/s41467-017-01327-4 29109438 PMC5673889

[B21] Li Y. Chen Z. Wu L. Tao W. (2020). Novel tumor mutation score versus tumor mutation burden in predicting survival after immunotherapy in pan-cancer patients from the MSK-IMPACT cohort. Ann. Transl. Med. 8:446. 10.21037/atm.2020.03.163 32395490 PMC7210182

[B22] Linehan W. M. Ricketts C. J. (2019). The Cancer Genome Atlas of renal cell carcinoma: findings and clinical implications. Nat. Rev. Urol. 16, 539–552. 10.1038/s41585-019-0211-5 31278395

[B23] Marchesi M. Andersson E. Villabona L. Seliger B. Lundqvist A. Kiessling R.. (2013). HLA-dependent tumour development: a role for tumour associate macrophages. J. Transl. Med. 11:247. 10.1186/1479-5876-11-247 24093459 PMC3856519

[B24] Motzer R. J. Hutson T. E. Cella D. Reeves J. Hawkins R. Guo J.. (2013). Pazopanib versus sunitinib in metastatic renal-cell carcinoma. N. Engl. J. Med. 369, 722–731. 10.1056/NEJMoa1303989 23964934

[B25] Peña-Llopis S. Vega-Rubín-de-Celis S. Liao A. Leng N. A. Pavía-Jiménez Wang S.. (2012). BAP1 loss defines a new class of renal cell carcinoma. Nat. Genet. 44, 751–759. 10.1038/ng.2323 22683710 PMC3788680

[B26] Pollard J. W. (2004). Tumour-educated macrophages promote tumour progression and metastasis. Nat. Rev. Cancer 4, 71–78. 10.1038/nrc1256 14708027

[B27] Pripp A. H. (2018). [Pearson's or Spearman's correlation coefficients]. Tidsskr. Nor. Laegeforen. 138:42. 10.4045/tidsskr.18.0042 29737766

[B28] Stickel J. S. Weinzierl A. O. Hillen N. Drews O. Schuler M. M. Hennenlotter J.. (2009). HLA ligand profiles of primary renal cell carcinoma maintained in metastases. Cancer Immunol. Immunother. 58, 1407–1417. 10.1007/s00262-008-0655-6 19184600 PMC11031011

[B29] Uhlén M. Fagerberg L. Hallström B. M. Lindskog C. Oksvold P. Mardinoglu A.. (2015). Proteomics. Tissue-based map of the human proteome. Science 347:1260419. 10.1126/science.1260419 25613900

[B30] Wang C. Wang Y. Hong T. Ye J. Chu C. Zuo L.. (2020). Targeting a positive regulatory loop in the tumor-macrophage interaction impairs the progression of clear cell renal cell carcinoma. Cell Death Differ. 10.1038/s41418-020-00626-6 33009518 PMC7937678

[B31] Wang Y. Roche O. Yan M. S. Finak G. Evans A. J. Metcalf J. L.. (2009). Regulation of endocytosis via the oxygen-sensing pathway. Nat. Med. 15, 319–324. 10.1038/nm.1922 19252501

[B32] Wang Y. Yan K. Lin J. Wang J. Zheng Z. Li X.. (2020). Three-gene risk model in papillary renal cell carcinoma: a robust likelihood-based survival analysis. Aging 12, 21854–21873. 10.18632/aging.104001 33154194 PMC7695399

[B33] Weinzierl A. O. Maurer D. Altenberend F. Schneiderhan-Marra N. Klingel K. Schoor O.. (2008). A cryptic vascular endothelial growth factor T-cell epitope: identification and characterization by mass spectrometry and T-cell assays. Cancer Res. 68, 2447–2454. 10.1158/0008-5472.CAN-07-2540 18381453

[B34] Xu L. Li Z. Song S. Chen Q. Mo L. Wang C.. (2020). Downregulation of α-l-fucosidase 1 suppresses glioma progression by enhancing autophagy and inhibiting macrophage infiltration. Cancer Sci. 111, 2284–2296. 10.1111/cas.14427 32314457 PMC7385365

[B35] Xu W. Atkins M. B. McDermott D. F. (2020). Checkpoint inhibitor immunotherapy in kidney cancer. Nat. Rev. Urol. 17, 137–150. 10.1038/s41585-020-0282-3 32020040

[B36] Yang Z. Wei S. Deng Y. Wang Z. Liu L. (2020). Clinical significance of tumour mutation burden in immunotherapy across multiple cancer types: an individual meta-analysis. Jpn. J. Clin. Oncol. 50, 1023–1031. 10.1093/jjco/hyaa076 32542383

[B37] Yoshihara K. Shahmoradgoli M. Martínez E. Vegesna R. Kim H.. (2013). Inferring tumour purity and stromal and immune cell admixture from expression data. Nat. Commun. 4:2612. 10.1038/ncomms3612 24113773 PMC3826632

