# Supplementary material for: Macrophage M2 Co-expression Factors Correlate With the Immune Microenvironment and Predict Outcome of Renal Clear Cell Carcinoma
Source: Front Genet. 2021 Feb 22;12:615655. doi: 10.3389/fgene.2021.615655 (PMC7938896; doi:10.3389/fgene.2021.615655)
Supplement: Supplementary Table 1 — The detail information of tumor mutation, tumor purity, and M2 macrophage proportion. [file Table_1.docx]

Supplementary Table 1

| **ID** | **Monocytes** | **Macrophages M0** | **MacrophagesM1** | **MacrophagesM2** | **M2/M1** |
| --- | --- | --- | --- | --- | --- |
| TCGA.A3.3323.01A.02R.1325.07 | 0.129332 | 0 | 0.113933 | 0.272837 | 2.394708 |
| TCGA.CJ.4904.01A.02R.1426.07 | 0.036693 | 0 | 0.183261 | 0.164317 | 0.896629 |
| TCGA.A3.3387.01A.01R.1541.07 | 0.083411 | 0 | 0.01964 | 0.385493 | 19.6283 |
| TCGA.A3.3326.01A.01R.0864.07 | 0.043902 | 0.0086 | 0.097853 | 0.141325 | 1.444261 |
| TCGA.CZ.4857.01A.01R.1305.07 | 0.115345 | 0.044751 | 0.068056 | 0.250945 | 3.687316 |
| TCGA.B0.5400.01A.01R.1503.07 | 0 | 0.167676 | 0.048947 | 0.228898 | 4.676432 |
| TCGA.BP.5198.01A.01R.1426.07 | 0 | 0.264189 | 0.027544 | 0.155603 | 5.649295 |
| TCGA.A3.3329.01A.01R.0864.07 | 0.071287 | 0 | 0.129703 | 0.239976 | 1.850193 |
| TCGA.B8.5550.01A.01R.1541.07 | 0.000139 | 0.165228 | 0.0839 | 0.269338 | 3.21023 |
| TCGA.BP.5010.01A.02R.1420.07 | 0 | 0.179701 | 0.082318 | 0.131523 | 1.597745 |
| TCGA.CJ.4644.01A.02R.1325.07 | 0.089291 | 0 | 0.086317 | 0.210067 | 2.433683 |
| TCGA.BP.4968.01A.01R.1334.07 | 0.007497 | 0.026208 | 0.076865 | 0.073007 | 0.94981 |
| TCGA.CJ.4872.01A.01R.1305.07 | 0.032566 | 0.031525 | 0.068568 | 0.09525 | 1.389143 |
| TCGA.BP.5009.01A.01R.1334.07 | 0.082959 | 0 | 0.065291 | 0.198089 | 3.033961 |
| TCGA.B0.4699.01A.01R.1277.07 | 0 | 0.033888 | 0.055299 | 0.16784 | 3.035153 |
| TCGA.BP.4998.01A.01R.1334.07 | 0.076944 | 0 | 0.061746 | 0.273597 | 4.431001 |
| TCGA.BP.5199.01A.01R.1426.07 | 0.005281 | 0.031201 | 0.056738 | 0.070407 | 1.240918 |
| TCGA.BP.4973.01A.01R.1334.07 | 0.031387 | 0 | 0.097369 | 0.131695 | 1.35254 |
| TCGA.B2.5635.01A.01R.A277.07 | 0.161622 | 0 | 0.040942 | 0.270408 | 6.604686 |
| TCGA.BP.5177.01A.01R.1426.07 | 0 | 0.098484 | 0.10854 | 0.082843 | 0.763251 |
| TCGA.B0.5707.01A.11R.1541.07 | 0.012359 | 0.163455 | 0.010238 | 0.320286 | 31.28372 |
| TCGA.BP.4971.01A.01R.1334.07 | 0.01717 | 0.190378 | 0.101071 | 0.123531 | 1.222216 |
| TCGA.B8.4620.01A.02R.1325.07 | 0.078018 | 0.11953 | 0.020641 | 0.208391 | 10.09618 |
| TCGA.BP.4801.01A.02R.1420.07 | 0.012063 | 0.08502 | 0.066777 | 0.433252 | 6.488077 |
| TCGA.B8.5158.01A.01R.1420.07 | 0.038731 | 0.031845 | 0.142736 | 0.105126 | 0.736508 |
| TCGA.A3.3308.01A.02R.1325.07 | 0.075338 | 0.040396 | 0.113104 | 0.346096 | 3.059985 |
| TCGA.BP.4807.01A.01R.1305.07 | 0.063947 | 0.000171 | 0.047979 | 0.510881 | 10.64792 |
| TCGA.A3.3378.01A.02R.1325.07 | 0.007262 | 0 | 0.074144 | 0.30234 | 4.077721 |
| TCGA.CJ.4869.01A.02R.1426.07 | 0.026436 | 0 | 0.043746 | 0.073722 | 1.685254 |
| TCGA.CJ.4895.01A.01R.1305.07 | 0.042712 | 0.062488 | 0.103473 | 0.220346 | 2.129512 |
| TCGA.B0.5691.01A.11R.1541.07 | 0.043464 | 0.043879 | 0.140348 | 0.126788 | 0.903387 |
| TCGA.BP.4781.01A.01R.1305.07 | 0.06606 | 0.137867 | 0.06614 | 0.309425 | 4.678372 |
| TCGA.CZ.5463.01A.01R.1503.07 | 0.033089 | 0 | 0.180613 | 0.221252 | 1.22501 |
| TCGA.6D.AA2E.01A.11R.A37O.07 | 0.028225 | 0 | 0.012997 | 0.13311 | 10.24174 |
| TCGA.B0.5711.01A.11R.1672.07 | 0.060942 | 0.077776 | 0.084841 | 0.123868 | 1.460001 |
| TCGA.B0.5088.01A.01R.1334.07 | 0 | 0.092775 | 0.053067 | 0.27171 | 5.120107 |
| TCGA.A3.3324.01A.02R.1325.07 | 0.08857 | 0 | 0.087035 | 0.211376 | 2.428643 |
| TCGA.BP.4981.01A.01R.1334.07 | 0.056865 | 0.002282 | 0.088465 | 0.175351 | 1.982148 |
| TCGA.CJ.4643.01A.02R.1325.07 | 0.09306 | 0 | 0.131588 | 0.13769 | 1.046371 |
| TCGA.BP.4977.01A.01R.1334.07 | 0.041742 | 0.04343 | 0.109014 | 0.240555 | 2.206651 |
| TCGA.A3.3362.01A.02R.1325.07 | 0.083979 | 0 | 0.096938 | 0.249305 | 2.571815 |
| TCGA.B0.5120.01A.01R.1420.07 | 0.014929 | 0.012003 | 0.055586 | 0.362236 | 6.516686 |
| TCGA.CJ.4916.01A.01R.1426.07 | 0.023477 | 0 | 0.080417 | 0.131634 | 1.636901 |
| TCGA.BP.4964.01A.01R.1334.07 | 0.006744 | 0.03061 | 0.137235 | 0.177057 | 1.290172 |
| TCGA.B0.4821.01A.01R.1503.07 | 0 | 0.320269 | 0.056771 | 0.041553 | 0.73194 |
| TCGA.CZ.4860.01A.01R.1305.07 | 0.004653 | 0.074423 | 0.109603 | 0.213261 | 1.945766 |
| TCGA.B0.4813.01A.01R.1277.07 | 0.028931 | 0.134574 | 0.061553 | 0.315335 | 5.122941 |
| TCGA.CJ.4892.01A.01R.1305.07 | 0.029967 | 0 | 0.045223 | 0.063488 | 1.403904 |
| TCGA.CW.5584.01A.01R.1541.07 | 0.213333 | 0 | 0.111845 | 0.196471 | 1.756633 |
| TCGA.BP.5180.01A.01R.1426.07 | 0.110046 | 0.043437 | 0.081838 | 0.221441 | 2.705846 |
| TCGA.CJ.4639.01A.02R.1325.07 | 0.010318 | 0 | 0.078008 | 0.073046 | 0.936391 |
| TCGA.BP.4782.01A.02R.1420.07 | 0.009309 | 0.049238 | 0.073036 | 0.288569 | 3.951055 |
| TCGA.CJ.4918.01A.01R.1426.07 | 0.014656 | 0.100928 | 0.059816 | 0.199266 | 3.331284 |
| TCGA.A3.3372.01A.02R.1325.07 | 0.080844 | 0 | 0.164123 | 0.146061 | 0.889945 |
| TCGA.B8.5165.01A.01R.1420.07 | 0.011291 | 0.009303 | 0.068724 | 0.199021 | 2.895942 |
| TCGA.A3.3343.01A.01R.0864.07 | 0.071022 | 0.028946 | 0.122892 | 0.164533 | 1.338839 |
| TCGA.B8.4154.01A.01R.1188.07 | 0.039225 | 0 | 0.135539 | 0.203423 | 1.50085 |
| TCGA.CJ.6030.01A.11R.1672.07 | 0.075142 | 0.001181 | 0.067833 | 0.253582 | 3.738356 |
| TCGA.B0.4842.01A.02R.1420.07 | 0.016625 | 0.13287 | 0.061168 | 0.097288 | 1.590511 |
| TCGA.A3.3349.01A.01R.1188.07 | 0.078497 | 0 | 0.023415 | 0.287442 | 12.27585 |
| TCGA.BP.4353.01A.02R.1289.07 | 0.004883 | 0 | 0.063839 | 0.427617 | 6.698396 |
| TCGA.B0.5077.01A.01R.1334.07 | 0.002103 | 0.226745 | 0.067994 | 0.137644 | 2.024335 |
| TCGA.CZ.5984.01A.11R.1672.07 | 0.032174 | 0 | 0.053612 | 0.135811 | 2.533218 |
| TCGA.B8.A54G.01A.11R.A266.07 | 0.066865 | 0 | 0.065711 | 0.158713 | 2.415326 |
| TCGA.A3.3346.01A.01R.1766.07 | 0.032366 | 0.115636 | 0.083825 | 0.205323 | 2.449417 |
| TCGA.B2.5633.01A.01R.1541.07 | 0.056358 | 0.061042 | 0.079631 | 0.301435 | 3.785417 |
| TCGA.BP.5173.01A.01R.1426.07 | 0.009107 | 0.003823 | 0.09325 | 0.115689 | 1.24063 |
| TCGA.BP.5007.01A.01R.1334.07 | 0.058397 | 0 | 0.113823 | 0.200203 | 1.758906 |
| TCGA.B0.5108.01A.01R.1420.07 | 0.015489 | 0.195403 | 0.078619 | 0.185037 | 2.353608 |
| TCGA.B0.5699.01A.11R.1541.07 | 0.091116 | 0.00091 | 0.136593 | 0.157931 | 1.156214 |
| TCGA.AK.3425.01A.02R.1277.07 | 0 | 0.086077 | 0.079183 | 0.255385 | 3.225244 |
| TCGA.AS.3778.01A.01R.A32Z.07 | 0.076781 | 0 | 0.133223 | 0.296853 | 2.22824 |
| TCGA.CW.6087.01A.11R.1672.07 | 0.04526 | 0 | 0.117583 | 0.15001 | 1.275785 |
| TCGA.BP.5170.01A.01R.1426.07 | 0.000621 | 0.053015 | 0.030119 | 0.341511 | 11.33887 |
| TCGA.B0.5121.01A.02R.1420.07 | 0.110509 | 0.005983 | 0.058458 | 0.082978 | 1.419454 |
| TCGA.CZ.5452.01A.01R.1503.07 | 0.04917 | 0 | 0.047917 | 0.106724 | 2.227281 |
| TCGA.A3.3316.01A.01R.0864.07 | 0.114643 | 0.153685 | 0.043254 | 0.386604 | 8.938081 |
| TCGA.B0.5095.01A.01R.1420.07 | 0.033917 | 0.062373 | 0.090552 | 0.20815 | 2.298671 |
| TCGA.BP.4972.01A.01R.1334.07 | 0.011358 | 0.109718 | 0.093442 | 0.397891 | 4.258154 |
| TCGA.B0.4712.01A.01R.1503.07 | 0.019603 | 0.086157 | 0.123505 | 0.171331 | 1.387239 |
| TCGA.CZ.5454.01A.01R.1503.07 | 0 | 0.182784 | 0.017862 | 0.45181 | 25.29475 |
| TCGA.B8.4148.01A.02R.1325.07 | 0.039897 | 0 | 0.096862 | 0.081988 | 0.846441 |
| TCGA.BP.4959.01A.01R.1334.07 | 0.055785 | 0.112273 | 0.020083 | 0.300418 | 14.95911 |
| TCGA.CZ.4858.01A.01R.1305.07 | 0.010176 | 0.057133 | 0.07659 | 0.14709 | 1.920487 |
| TCGA.CW.6090.01A.11R.1672.07 | 0.031246 | 0.088629 | 0.029885 | 0.347648 | 11.6328 |
| TCGA.CZ.5470.01A.01R.1503.07 | 0.083079 | 0 | 0.085622 | 0.145957 | 1.704669 |
| TCGA.B0.5097.01A.01R.1420.07 | 0 | 0.313871 | 0.052564 | 0.192593 | 3.663978 |
| TCGA.BP.4162.01A.02R.1325.07 | 0.00601 | 0 | 0.092071 | 0.170348 | 1.850184 |
| TCGA.BP.4343.01A.02R.1289.07 | 0.048366 | 0.039036 | 0.122396 | 0.266001 | 2.173287 |
| TCGA.AK.3458.01A.01R.1503.07 | 0.008629 | 0 | 0.068866 | 0.150009 | 2.178267 |
| TCGA.CZ.5457.01A.01R.1503.07 | 0.038132 | 0 | 0.082548 | 0.264422 | 3.203232 |
| TCGA.BP.4331.01A.01R.1289.07 | 0.176776 | 0 | 0.064401 | 0.254607 | 3.953483 |
| TCGA.BP.4787.01A.01R.1305.07 | 0.100358 | 0.012525 | 0.07403 | 0.357176 | 4.824771 |
| TCGA.BP.4795.01A.02R.1420.07 | 0.050722 | 0 | 0.026985 | 0.16212 | 6.007787 |
| TCGA.AK.3451.01A.02R.1188.07 | 0.040939 | 0.038166 | 0.046432 | 0.189847 | 4.088721 |
| TCGA.MM.A564.01A.11R.A266.07 | 0.178338 | 0.036071 | 0.040779 | 0.128226 | 3.144411 |
| TCGA.CJ.4901.01A.01R.1426.07 | 0 | 0.032204 | 0.106003 | 0.069771 | 0.658199 |
| TCGA.B0.5092.01A.01R.1420.07 | 0.002267 | 0 | 0.068917 | 0.077979 | 1.131491 |
| TCGA.BP.4160.01A.02R.1289.07 | 0.011231 | 0 | 0.070334 | 0.10996 | 1.563402 |
| TCGA.B2.3924.01A.02R.A277.07 | 0.060319 | 0 | 0.133973 | 0.135588 | 1.01205 |
| TCGA.BP.4173.01A.02R.1289.07 | 0.07711 | 0 | 0.073231 | 0.106238 | 1.450712 |
| TCGA.B2.5635.01A.01R.1541.07 | 0.142015 | 0 | 0.071108 | 0.172614 | 2.427494 |
| TCGA.AK.3434.01A.02R.1277.07 | 0 | 0.021337 | 0.155207 | 0.119609 | 0.770641 |
| TCGA.BP.4989.01A.01R.1334.07 | 0.010178 | 0.002998 | 0.043168 | 0.069681 | 1.614195 |
| TCGA.B2.A4SR.01A.11R.A266.07 | 0.014395 | 0.010723 | 0.123971 | 0.178482 | 1.439704 |
| TCGA.B0.5102.01A.01R.1420.07 | 0.004682 | 0.028913 | 0.088434 | 0.238515 | 2.697112 |
| TCGA.BP.4167.01A.02R.1325.07 | 0.009602 | 0.008683 | 0.059288 | 0.254869 | 4.298859 |
| TCGA.CZ.5462.01A.01R.1503.07 | 0.154227 | 0.037235 | 0.117995 | 0.143322 | 1.21464 |
| TCGA.G6.A5PC.01A.11R.A33J.07 | 0.053336 | 0 | 0.062734 | 0.108431 | 1.728424 |
| TCGA.B2.4099.01A.02R.1188.07 | 0.128972 | 0 | 0.125379 | 0.313512 | 2.500521 |
| TCGA.A3.3365.01A.01R.0864.07 | 0.010256 | 0 | 0.09394 | 0.19402 | 2.06537 |
| TCGA.B0.4706.01A.01R.1503.07 | 0.043811 | 0.006283 | 0.071581 | 0.033716 | 0.471011 |
| TCGA.CJ.4920.01A.01R.1426.07 | 0.100286 | 0.076233 | 0.060754 | 0.237761 | 3.913499 |
| TCGA.B8.A54I.01A.21R.A33J.07 | 0.01303 | 0 | 0.003458 | 0.188233 | 54.42928 |
| TCGA.CJ.4893.01A.01R.1305.07 | 0.056655 | 0 | 0.077446 | 0.12934 | 1.670058 |
| TCGA.CJ.4882.01A.02R.1426.07 | 0.003971 | 0.028932 | 0.060247 | 0.063383 | 1.052056 |
| TCGA.B0.5709.01A.11R.1541.07 | 0.074097 | 0 | 0.101618 | 0.125087 | 1.230963 |
| TCGA.B0.5706.01A.11R.1541.07 | 0.003877 | 0.026758 | 0.037959 | 0.045293 | 1.193214 |
| TCGA.B2.3924.01A.02R.1325.07 | 0.050038 | 0 | 0.078226 | 0.153787 | 1.965928 |
| TCGA.CJ.4902.01A.01R.1426.07 | 0.021424 | 0.062521 | 0.086993 | 0.199155 | 2.28932 |
| TCGA.BP.4963.01A.01R.1334.07 | 0.116868 | 0.026391 | 0.094806 | 0.241419 | 2.546458 |
| TCGA.AK.3460.01A.02R.1277.07 | 0.075752 | 0 | 0.059354 | 0.320597 | 5.401411 |
| TCGA.AK.3453.01A.02R.1277.07 | 0.032505 | 0 | 0.067787 | 0.050833 | 0.749883 |
| TCGA.B0.5697.01A.11R.1541.07 | 0.016064 | 0 | 0.049282 | 0.177823 | 3.60826 |
| TCGA.B0.4841.01A.01R.1277.07 | 0.043467 | 0.004067 | 0.107452 | 0.20795 | 1.935284 |
| TCGA.CJ.4891.01A.01R.1305.07 | 0.18785 | 0.108473 | 0.045015 | 0.227672 | 5.057712 |
| TCGA.B0.4818.01A.01R.1503.07 | 0.036923 | 0 | 0.074 | 0.032988 | 0.445779 |
| TCGA.B0.4714.01A.01R.1277.07 | 0.019551 | 0 | 0.105709 | 0.135492 | 1.281745 |
| TCGA.A3.3374.01A.02R.1325.07 | 0 | 0.701937 | 0.005604 | 0.193549 | 34.53452 |
| TCGA.A3.3373.01A.02R.1420.07 | 0.049723 | 0.02088 | 0.117151 | 0.250194 | 2.135664 |
| TCGA.CJ.5679.01A.11R.1541.07 | 0 | 0.490269 | 0.017681 | 0.102386 | 5.790756 |
| TCGA.BP.4798.01A.01R.1305.07 | 0.089215 | 0 | 0.087206 | 0.130771 | 1.499564 |
| TCGA.B8.5549.01A.01R.1541.07 | 0.050747 | 0.00287 | 0.073811 | 0.153989 | 2.086258 |
| TCGA.DV.5574.01A.01R.1541.07 | 0.022371 | 0.048926 | 0.052579 | 0.258539 | 4.917156 |
| TCGA.BP.5194.01A.02R.1426.07 | 0.014803 | 0.023296 | 0.095108 | 0.186627 | 1.962253 |
| TCGA.B8.5545.01A.01R.1672.07 | 0.025143 | 0 | 0.081097 | 0.291454 | 3.59389 |
| TCGA.B0.4846.01A.01R.1277.07 | 0.01292 | 0 | 0.156828 | 0.148491 | 0.946839 |
| TCGA.CJ.4876.01A.01R.1305.07 | 0.012061 | 0 | 0.072893 | 0.038924 | 0.533984 |
| TCGA.BP.4345.01A.01R.1289.07 | 0.009789 | 0.329336 | 0.057317 | 0.174492 | 3.04435 |
| TCGA.B0.4838.01A.01R.1305.07 | 0.011946 | 0.000142 | 0.058139 | 0.030613 | 0.526541 |
| TCGA.B0.5075.01A.01R.1334.07 | 0.015088 | 0.162064 | 0.028671 | 0.372053 | 12.97642 |
| TCGA.CZ.5458.01A.01R.1503.07 | 0.083243 | 0 | 0.07161 | 0.145977 | 2.038496 |
| TCGA.B0.4690.01A.01R.1277.07 | 0.101579 | 0 | 0.101876 | 0.192408 | 1.88865 |
| TCGA.BP.4335.01A.01R.1289.07 | 0.005836 | 0 | 0.109009 | 0.094854 | 0.870152 |
| TCGA.A3.3347.01A.02R.1325.07 | 0 | 0.188626 | 0.076919 | 0.303721 | 3.948556 |
| TCGA.B0.4827.01A.02R.1420.07 | 0.030597 | 0 | 0.057086 | 0.171467 | 3.003685 |
| TCGA.B0.5402.01A.01R.1503.07 | 0.039637 | 0 | 0.19623 | 0.167043 | 0.85126 |
| TCGA.CW.6097.01A.11R.1672.07 | 0.022365 | 0.012144 | 0.06258 | 0.142277 | 2.27352 |
| TCGA.A3.A6NL.01A.11R.A33J.07 | 0.065561 | 0.014709 | 0.0832 | 0.137671 | 1.654703 |
| TCGA.CJ.5689.01A.11R.1541.07 | 0.020384 | 0.038643 | 0.027031 | 0.245715 | 9.090195 |
| TCGA.A3.3307.01A.01R.0864.07 | 0.141719 | 0 | 0.109139 | 0.224965 | 2.061269 |
| TCGA.CZ.4862.01A.01R.1305.07 | 0.035018 | 0 | 0.115106 | 0.115658 | 1.004801 |
| TCGA.CJ.5675.01A.11R.1541.07 | 0.002099 | 0 | 0.054261 | 0.08972 | 1.653498 |
| TCGA.CJ.4886.01A.01R.1305.07 | 0.020436 | 0 | 0.10942 | 0.211024 | 1.928565 |
| TCGA.B0.5106.01A.01R.1420.07 | 0.052927 | 0 | 0.038596 | 0.159043 | 4.120713 |
| TCGA.B0.4843.01A.01R.1277.07 | 0.040708 | 0.231615 | 0.048 | 0.161765 | 3.370095 |
| TCGA.B0.5083.01A.02R.1420.07 | 0.027596 | 0.170226 | 0.054993 | 0.307488 | 5.591382 |
| TCGA.BP.4325.01A.02R.1289.07 | 0.023129 | 0 | 0.078028 | 0.161752 | 2.073016 |
| TCGA.CW.5580.01A.01R.1672.07 | 0.215061 | 0 | 0.086665 | 0.331765 | 3.82813 |
| TCGA.BP.5000.01A.01R.1334.07 | 0.047885 | 0 | 0.095937 | 0.133552 | 1.392084 |
| TCGA.B8.A54H.01A.11R.A33J.07 | 0.025073 | 0 | 0.029016 | 0.092046 | 3.172253 |
| TCGA.B0.4701.01A.01R.1277.07 | 0.029069 | 0.011609 | 0.103694 | 0.106886 | 1.03078 |
| TCGA.BP.4161.01A.02R.1325.07 | 0.014241 | 0.021878 | 0.088581 | 0.190908 | 2.155186 |
| TCGA.B8.4143.01A.01R.1188.07 | 0.011573 | 0.027927 | 0.09313 | 0.165069 | 1.772456 |
| TCGA.B0.4847.01A.01R.1277.07 | 0.031033 | 0 | 0.067656 | 0.051372 | 0.759316 |
| TCGA.CZ.5985.01A.11R.1672.07 | 0.008734 | 0.057574 | 0.094399 | 0.251644 | 2.665755 |
| TCGA.CZ.4865.01A.02R.1503.07 | 0.025075 | 0.041571 | 0.077953 | 0.189577 | 2.431931 |
| TCGA.A3.3359.01A.01R.0864.07 | 0.044625 | 0 | 0.138482 | 0.164288 | 1.186353 |
| TCGA.CJ.6031.01A.11R.1672.07 | 0 | 0.098203 | 0.06371 | 0.21456 | 3.367778 |
| TCGA.AK.3454.01A.02R.1277.07 | 0.009759 | 0 | 0.042093 | 0.262361 | 6.232887 |
| TCGA.CJ.5684.01A.11R.1541.07 | 0.002481 | 0.007833 | 0.063705 | 0.139467 | 2.189257 |
| TCGA.CJ.4900.01A.01R.1334.07 | 0.032562 | 0.017235 | 0.0797 | 0.121067 | 1.519028 |
| TCGA.B0.5113.01A.01R.1420.07 | 0.00855 | 0 | 0.106894 | 0.148075 | 1.385247 |
| TCGA.CJ.4908.01A.01R.1426.07 | 0.052207 | 0.01209 | 0.086855 | 0.246656 | 2.839853 |
| TCGA.CZ.4866.01A.01R.1503.07 | 0.100905 | 0.099559 | 0.053818 | 0.414264 | 7.697504 |
| TCGA.B8.4621.01A.01R.1503.07 | 0.0945 | 0.06793 | 0.035141 | 0.410812 | 11.6903 |
| TCGA.B0.4697.01A.01R.1277.07 | 0.000371 | 0.031893 | 0.090812 | 0.04763 | 0.52449 |
| TCGA.B0.5690.01A.11R.1541.07 | 0.097403 | 0.059445 | 0.094796 | 0.114639 | 1.209327 |
| TCGA.BP.4992.01A.01R.1334.07 | 0.020304 | 0.009393 | 0.071223 | 0.043932 | 0.616823 |
| TCGA.B0.4691.01A.01R.1277.07 | 0.01932 | 0 | 0.095058 | 0.076909 | 0.809072 |
| TCGA.B0.4703.01A.01R.1277.07 | 0.010021 | 0.034432 | 0.116271 | 0.192399 | 1.654746 |
| TCGA.A3.3380.01A.01R.0864.07 | 0.018667 | 0.012026 | 0.060756 | 0.308772 | 5.082154 |
| TCGA.B8.4622.01A.02R.1277.07 | 0.137529 | 0 | 0.062219 | 0.26587 | 4.273126 |
| TCGA.A3.3317.01A.02R.1325.07 | 0.032398 | 0.022326 | 0.141786 | 0.259863 | 1.832778 |
| TCGA.BP.5196.01A.01R.1426.07 | 0 | 0.079608 | 0.069135 | 0.125638 | 1.817289 |
| TCGA.B8.A54D.01A.21R.A266.07 | 0.016531 | 0 | 0.034532 | 0.180302 | 5.221345 |
| TCGA.B0.5702.01A.11R.1541.07 | 0.046555 | 0.001798 | 0.021936 | 0.250407 | 11.41548 |
| TCGA.DV.5573.01A.01R.1541.07 | 0.017928 | 0 | 0.040644 | 0.064497 | 1.586869 |
| TCGA.CJ.5672.01A.11R.1541.07 | 0.035606 | 0 | 0.075156 | 0.102778 | 1.367518 |
| TCGA.CJ.5677.01A.11R.1541.07 | 0.019756 | 0.317401 | 0.031293 | 0.296171 | 9.46446 |
| TCGA.A3.3351.01A.02R.1325.07 | 0.12211 | 0 | 0.069364 | 0.20458 | 2.949358 |
| TCGA.BP.4975.01A.01R.1334.07 | 0.05915 | 0 | 0.042589 | 0.312873 | 7.346356 |
| TCGA.BP.4346.01A.01R.1289.07 | 0.045382 | 0 | 0.063951 | 0.078746 | 1.231346 |
| TCGA.B0.4839.01A.01R.1305.07 | 0.102041 | 0 | 0.038325 | 0.190657 | 4.974714 |
| TCGA.CJ.5678.01A.11R.1541.07 | 0.025061 | 0 | 0.076078 | 0.118986 | 1.563991 |
| TCGA.AK.3429.01A.02R.1325.07 | 0.00644 | 0 | 0.098455 | 0.050373 | 0.511636 |
| TCGA.BP.4760.01A.02R.1420.07 | 0 | 0.511432 | 0.032612 | 0.141983 | 4.353664 |
| TCGA.AK.3445.01A.02R.1277.07 | 0.002847 | 0.010435 | 0.106676 | 0.244315 | 2.290258 |
| TCGA.BP.5004.01A.01R.1334.07 | 0 | 0.354254 | 0.047895 | 0.249604 | 5.211517 |
| TCGA.BP.4159.01A.02R.1289.07 | 0.101752 | 0.043832 | 0.08868 | 0.194669 | 2.195195 |
| TCGA.BP.4970.01A.01R.1334.07 | 0.112408 | 0 | 0.068532 | 0.218528 | 3.188694 |
| TCGA.BP.4330.01A.01R.1289.07 | 0.035211 | 0 | 0.091929 | 0.09044 | 0.983803 |
| TCGA.BP.4962.01A.01R.1334.07 | 0.066431 | 0.009715 | 0.063237 | 0.180025 | 2.846823 |
| TCGA.CJ.4889.01A.01R.1305.07 | 0.007962 | 0 | 0.10515 | 0.143517 | 1.364875 |
| TCGA.CJ.4899.01A.01R.1334.07 | 0.014774 | 0.011484 | 0.049599 | 0.408728 | 8.240722 |
| TCGA.BP.4174.01A.02R.1289.07 | 0.060127 | 0.018633 | 0.111912 | 0.227509 | 2.032926 |
| TCGA.CJ.5682.01A.11R.1541.07 | 0.021499 | 3.54E-05 | 0.105578 | 0.073216 | 0.693474 |
| TCGA.BP.4771.01A.01R.1289.07 | 0.022599 | 0 | 0.060025 | 0.062045 | 1.033649 |
| TCGA.BP.4803.01A.01R.1305.07 | 0.058799 | 0.057291 | 0.082753 | 0.278746 | 3.368423 |
| TCGA.CJ.4897.01A.03R.1426.07 | 0.027578 | 0.028652 | 0.094509 | 0.137805 | 1.458109 |
| TCGA.B0.5107.01A.01R.1420.07 | 0.050548 | 0 | 0.128383 | 0.119634 | 0.931853 |
| TCGA.BP.4759.01A.01R.1289.07 | 0.032445 | 0.041398 | 0.074295 | 0.164057 | 2.208194 |
| TCGA.CJ.5671.01A.11R.1541.07 | 0.03721 | 0.182862 | 0.046925 | 0.203773 | 4.342497 |
| TCGA.B0.4836.01A.01R.1305.07 | 0 | 0.116737 | 0.108623 | 0.061088 | 0.562389 |
| TCGA.CZ.4864.01A.01R.1503.07 | 0.052067 | 0 | 0.098473 | 0.188655 | 1.915805 |
| TCGA.AK.3436.01A.02R.1325.07 | 0 | 0.125428 | 0.03669 | 0.180681 | 4.924559 |
| TCGA.A3.3382.01A.02R.1325.07 | 0.00487 | 0.024112 | 0.086334 | 0.297192 | 3.442336 |
| TCGA.CZ.5464.01A.01R.1503.07 | 0.052545 | 0.042997 | 0.076116 | 0.164021 | 2.154886 |
| TCGA.BP.5186.01A.01R.1426.07 | 0.011261 | 0 | 0.067068 | 0.095977 | 1.43104 |
| TCGA.CZ.4856.01A.02R.1426.07 | 0.011488 | 0 | 0.106309 | 0.132661 | 1.247879 |
| TCGA.A3.3376.01A.02R.1420.07 | 0.204473 | 0 | 0.022914 | 0.274247 | 11.96855 |
| TCGA.CJ.4635.01A.02R.1305.07 | 0.00634 | 0.10167 | 0.032641 | 0.208675 | 6.392975 |
| TCGA.BP.5183.01A.01R.1426.07 | 0.012273 | 0 | 0.091739 | 0.141718 | 1.544795 |
| TCGA.B8.5163.01A.01R.1420.07 | 0.037029 | 0 | 0.079953 | 0.224364 | 2.80619 |
| TCGA.CZ.5461.01A.01R.1503.07 | 0.098897 | 0 | 0.120601 | 0.196273 | 1.627459 |
| TCGA.B0.5701.01A.11R.1541.07 | 0.025933 | 0.116979 | 0.10051 | 0.18183 | 1.809074 |
| TCGA.B2.5639.01A.01R.1541.07 | 0.12157 | 0 | 0.069389 | 0.145205 | 2.092607 |
| TCGA.B2.3924.01B.03R.A277.07 | 0 | 0 | 0.162901 | 0.171334 | 1.051772 |
| TCGA.BP.5181.01A.01R.1426.07 | 0.073886 | 0 | 0.113838 | 0.189208 | 1.662075 |
| TCGA.B2.5633.01A.01R.A277.07 | 0.118052 | 0.07153 | 0.05457 | 0.302843 | 5.549591 |
| TCGA.AK.3431.01A.02R.1277.07 | 0.074182 | 0.138008 | 0.049537 | 0.164842 | 3.327652 |
| TCGA.B0.4811.01A.01R.1503.07 | 0.020714 | 0.011198 | 0.162566 | 0.102836 | 0.632579 |
| TCGA.A3.A6NJ.01A.12R.A33J.07 | 0.032089 | 0 | 0.097229 | 0.066844 | 0.687498 |
| TCGA.BP.4797.01A.01R.1305.07 | 0.073132 | 0.037334 | 0.062968 | 0.214988 | 3.414236 |
| TCGA.B8.5553.01A.01R.1541.07 | 0.026515 | 0 | 0.080965 | 0.182432 | 2.253222 |
| TCGA.BP.4761.01A.01R.1289.07 | 0.031101 | 0.033638 | 0.06431 | 0.084964 | 1.321166 |
| TCGA.B0.4816.01A.01R.1503.07 | 0.251058 | 0 | 0.041072 | 0.252408 | 6.145579 |
| TCGA.CZ.4863.01A.01R.1503.07 | 0.001418 | 0 | 0.085646 | 0.088573 | 1.03418 |
| TCGA.CJ.4894.01A.01R.1305.07 | 0.053958 | 0 | 0.067238 | 0.090821 | 1.350741 |
| TCGA.CW.5588.01A.01R.1541.07 | 0.101903 | 0.07603 | 0.06148 | 0.329666 | 5.362146 |
| TCGA.A3.3325.01A.01R.0864.07 | 0.049166 | 0 | 0.040662 | 0.31657 | 7.785428 |
| TCGA.CJ.4871.01A.01R.1305.07 | 0.060419 | 0.053113 | 0.118541 | 0.2327 | 1.963035 |
| TCGA.BP.4804.01A.02R.1305.07 | 0 | 0.124542 | 0.09127 | 0.180495 | 1.977586 |
| TCGA.CJ.4873.01A.01R.1305.07 | 0.040748 | 0.028179 | 0.065223 | 0.248792 | 3.814495 |
| TCGA.CJ.4640.01A.02R.1325.07 | 0.077073 | 0 | 0.106697 | 0.176185 | 1.65126 |
| TCGA.BP.4762.01A.02R.1289.07 | 0.057296 | 0 | 0.0495 | 0.226651 | 4.578776 |
| TCGA.A3.3363.01A.01R.0864.07 | 0.05394 | 0.003593 | 0.00908 | 0.455509 | 50.16862 |
| TCGA.CZ.5468.01A.01R.1503.07 | 0 | 0.335403 | 0.016962 | 0.178602 | 10.52968 |
| TCGA.CZ.5466.01A.01R.1503.07 | 0.013147 | 0.10863 | 0.064858 | 0.200837 | 3.096553 |
| TCGA.CZ.4861.01A.01R.1305.07 | 0.094791 | 0.034518 | 0.109533 | 0.289158 | 2.639907 |
| TCGA.B0.4819.01A.01R.1277.07 | 0.001861 | 0.021806 | 0.065364 | 0.071691 | 1.096792 |
| TCGA.B0.5694.01A.11R.1541.07 | 0.06679 | 0.077376 | 0.078786 | 0.216054 | 2.742293 |
| TCGA.B0.5698.01A.11R.1672.07 | 0.089357 | 0.039735 | 0.097319 | 0.116431 | 1.196389 |
| TCGA.BP.5169.01A.01R.1426.07 | 0 | 0.076825 | 0.032148 | 0.059428 | 1.84856 |
| TCGA.B0.4700.01A.02R.1541.07 | 0 | 0.08459 | 0.106487 | 0.103238 | 0.969484 |
| TCGA.CJ.4875.01A.01R.1305.07 | 0.032051 | 0.016362 | 0.01835 | 0.068624 | 3.739721 |
| TCGA.B0.5115.01A.01R.1420.07 | 0.028574 | 0.035097 | 0.082874 | 0.155493 | 1.87626 |
| TCGA.B8.5552.01B.11R.1672.07 | 0.011079 | 0.131293 | 0.047409 | 0.275141 | 5.803595 |
| TCGA.CJ.6027.01A.11R.1672.07 | 0 | 0.088355 | 0.107432 | 0.149347 | 1.39015 |
| TCGA.CW.5589.01A.01R.1541.07 | 0.023975 | 0.071735 | 0.040095 | 0.359929 | 8.976883 |
| TCGA.BP.4987.01A.01R.1334.07 | 0.061495 | 0 | 0.0713 | 0.184271 | 2.58446 |
| TCGA.B2.5641.01A.01R.1541.07 | 0.053043 | 0.002943 | 0.059977 | 0.156959 | 2.61698 |
| TCGA.B0.4833.01A.01R.1305.07 | 0.018858 | 0.014812 | 0.078142 | 0.079635 | 1.019111 |
| TCGA.CJ.4874.01A.01R.1305.07 | 0.007686 | 0 | 0.128052 | 0.154962 | 1.210149 |
| TCGA.CJ.4887.01A.01R.1305.07 | 0.065251 | 0 | 0.080205 | 0.216506 | 2.699397 |
| TCGA.B8.5164.01A.01R.1420.07 | 0.023609 | 0 | 0.054691 | 0.115899 | 2.119152 |
| TCGA.AK.3428.01A.02R.1277.07 | 0.302083 | 0.02828 | 0.079309 | 0.065199 | 0.822098 |
| TCGA.B8.A54F.01A.11R.A266.07 | 0.224159 | 0 | 0.020329 | 0.346832 | 17.06057 |
| TCGA.BP.5187.01A.01R.1426.07 | 0.010947 | 0.012963 | 0.121774 | 0.099454 | 0.816708 |
| TCGA.CJ.5676.01A.11R.1541.07 | 0.01878 | 0 | 0.047451 | 0.245033 | 5.163883 |
| TCGA.DV.5565.01A.01R.1541.07 | 0.125281 | 0.099235 | 0.082574 | 0.20397 | 2.470137 |
| TCGA.CJ.5681.01A.11R.1541.07 | 0.049004 | 0.039267 | 0.004329 | 0.123705 | 28.57551 |
| TCGA.B8.A8YJ.01A.13R.A39I.07 | 0.061501 | 0 | 0.018406 | 0.193419 | 10.50872 |
| TCGA.B0.4707.01A.01R.1277.07 | 0.017496 | 0.017475 | 0.073059 | 0.016601 | 0.227223 |
| TCGA.BP.4770.01A.01R.1503.07 | 0 | 0.223646 | 0.028307 | 0.286867 | 10.13396 |
| TCGA.BP.4332.01A.01R.1289.07 | 0.061406 | 0.090239 | 0.030513 | 0.378477 | 12.40384 |
| TCGA.B0.4698.01A.01R.1503.07 | 0 | 0.039657 | 0.078152 | 0.12504 | 1.599949 |
| TCGA.BP.4982.01A.01R.1334.07 | 0.049315 | 0 | 0.073395 | 0.302566 | 4.12244 |
| TCGA.CW.5587.01A.01R.1541.07 | 0.060992 | 0 | 0.080228 | 0.201733 | 2.514488 |
| TCGA.BP.5201.01A.01R.1426.07 | 0.017859 | 0 | 0.090117 | 0.088285 | 0.979679 |
| TCGA.CW.5590.01A.01R.1541.07 | 0.027782 | 0.044309 | 0.138841 | 0.171801 | 1.237396 |
| TCGA.A3.3370.01A.02R.1420.07 | 0.020989 | 0 | 0.090136 | 0.189196 | 2.098992 |
| TCGA.BP.4166.01A.02R.1289.07 | 0.013729 | 0.030818 | 0.053527 | 0.195653 | 3.655246 |
| TCGA.B0.4810.01A.01R.1503.07 | 0.027482 | 0 | 0.073112 | 0.151533 | 2.072612 |
| TCGA.B0.4718.01A.01R.1277.07 | 0.023429 | 0 | 0.10698 | 0.131955 | 1.233447 |
| TCGA.B4.5834.01A.11R.1672.07 | 0.050692 | 0 | 0.186695 | 0.189998 | 1.017689 |
| TCGA.BP.4169.01A.02R.1289.07 | 0.079628 | 0.010399 | 0.149988 | 0.123334 | 0.822293 |
| TCGA.BP.4777.01A.01R.1289.07 | 0.015293 | 0.017824 | 0.050319 | 0.18205 | 3.617953 |
| TCGA.B0.4848.01A.01R.1277.07 | 0.09615 | 0.048013 | 0.110874 | 0.237353 | 2.140735 |
| TCGA.BP.4354.01A.02R.1289.07 | 0.06996 | 0.001211 | 0.075294 | 0.470082 | 6.243257 |
| TCGA.CJ.4642.01B.01R.1305.07 | 0 | 0.211033 | 0.044919 | 0.223428 | 4.974034 |
| TCGA.BP.5178.01A.01R.1426.07 | 0 | 0.130038 | 0.032345 | 0.257681 | 7.966548 |
| TCGA.BP.4790.01A.01R.1305.07 | 0.019071 | 0.066063 | 0.080965 | 0.114556 | 1.414876 |
| TCGA.B0.5399.01A.01R.1503.07 | 0.01499 | 0 | 0.107258 | 0.12086 | 1.126814 |
| TCGA.CJ.4641.01A.02R.1325.07 | 0.011103 | 0.009268 | 0.100918 | 0.123556 | 1.224313 |
| TCGA.BP.5195.01A.02R.1426.07 | 0.132537 | 0 | 0.081128 | 0.260181 | 3.207046 |
| TCGA.MM.A84U.01A.11R.A37O.07 | 0.066653 | 0 | 0.072205 | 0.189485 | 2.624276 |
| TCGA.CJ.4637.01A.02R.1325.07 | 0.057064 | 0 | 0.10938 | 0.084897 | 0.776165 |
| TCGA.CJ.4636.01A.02R.1325.07 | 0.038519 | 0 | 0.064131 | 0.300474 | 4.685312 |
| TCGA.AK.3455.01A.01R.0864.07 | 9.58E-05 | 0 | 0.054111 | 0.020611 | 0.380912 |
| TCGA.A3.A6NN.01A.12R.A33J.07 | 0.088598 | 0 | 0.081767 | 0.230594 | 2.820135 |
| TCGA.AK.3426.01A.02R.1325.07 | 0.012086 | 0.092127 | 0.065668 | 0.139387 | 2.122594 |
| TCGA.CJ.4890.01A.01R.1305.07 | 0.005812 | 0.046777 | 0.042937 | 0.199431 | 4.644694 |
| TCGA.EU.5906.01A.11R.1672.07 | 0.061987 | 0.01612 | 0.078485 | 0.258692 | 3.296054 |
| TCGA.BP.5182.01A.01R.1426.07 | 0.036779 | 0.09816 | 0.076467 | 0.271159 | 3.546076 |
| TCGA.DV.5575.01A.01R.1541.07 | 0.058568 | 0.005255 | 0.072648 | 0.183822 | 2.530326 |
| TCGA.B0.5703.01A.11R.1541.07 | 0.039317 | 0.023728 | 0.04167 | 0.004172 | 0.100118 |
| TCGA.BP.4176.01A.02R.1289.07 | 0.046017 | 0.139793 | 0.035593 | 0.278378 | 7.821246 |
| TCGA.B0.5692.01A.11R.1541.07 | 0.005983 | 0.001619 | 0.065572 | 0.031974 | 0.487621 |
| TCGA.BP.5175.01A.01R.1426.07 | 0.017966 | 0.084721 | 0.054698 | 0.176612 | 3.22887 |
| TCGA.DV.5569.01A.01R.1541.07 | 0.061364 | 0.059121 | 0.109985 | 0.176322 | 1.603147 |
| TCGA.BP.5189.01A.02R.1426.07 | 0.159478 | 0 | 0.075773 | 0.25075 | 3.309225 |
| TCGA.G6.A8L6.01A.11R.A37O.07 | 0.200671 | 0.010686 | 0.057596 | 0.150555 | 2.613965 |
| TCGA.B4.5838.01A.11R.1672.07 | 0.018939 | 0.058666 | 0.110086 | 0.204905 | 1.861308 |
| TCGA.BP.4342.01A.01R.1289.07 | 0.032641 | 0.021957 | 0.156843 | 0.155577 | 0.991923 |
| TCGA.BP.4163.01A.02R.1325.07 | 0.105503 | 0 | 0.123453 | 0.249869 | 2.024003 |
| TCGA.B0.5710.01A.11R.1672.07 | 0.014536 | 0.156094 | 0.109916 | 0.13451 | 1.223758 |
| TCGA.CJ.4884.01A.01R.1305.07 | 0.011585 | 0.023692 | 0.073392 | 0.204947 | 2.792505 |
| TCGA.CZ.5459.01A.01R.1503.07 | 0 | 0.028606 | 0.134809 | 0.175423 | 1.301274 |
| TCGA.B0.5096.01A.01R.1420.07 | 0.055584 | 0.196068 | 0.065606 | 0.296088 | 4.513132 |
| TCGA.B0.4837.01A.01R.1305.07 | 0 | 0.433484 | 0.002061 | 0.159979 | 77.63223 |
| TCGA.BP.4993.01A.02R.1420.07 | 0.021955 | 0 | 0.065617 | 0.156265 | 2.381465 |
| TCGA.DV.5568.01A.01R.1541.07 | 0 | 0.25825 | 0.05117 | 0.126796 | 2.477927 |
| TCGA.B0.4694.01A.01R.1277.07 | 0 | 0.123206 | 0.071345 | 0.18021 | 2.52589 |
| TCGA.B0.4823.01A.02R.1420.07 | 0.010354 | 0 | 0.040028 | 0.194005 | 4.846686 |
| TCGA.MM.A563.01A.11R.A266.07 | 0.065095 | 0.050041 | 0.107563 | 0.157829 | 1.467313 |
| TCGA.CJ.4868.01A.01R.1305.07 | 0.021372 | 0.099141 | 0.085221 | 0.210258 | 2.467203 |
| TCGA.AK.3456.01A.02R.1325.07 | 0.023544 | 0.093597 | 0.029948 | 0.443763 | 14.81768 |
| TCGA.B8.4146.01B.11R.1672.07 | 0.016585 | 0.028757 | 0.139407 | 0.122823 | 0.881038 |
