# Supplementary material for: Macrophage M2 Co-expression Factors Correlate With the Immune Microenvironment and Predict Outcome of Renal Clear Cell Carcinoma
Source: Front Genet. 2021 Feb 22;12:615655. doi: 10.3389/fgene.2021.615655 (PMC7938896; doi:10.3389/fgene.2021.615655)
Supplement: Supplementary Table 2 — The process data of multi-Cox regression risk score model. [file Table_2.docx]

| **Variables in the equation** | | | | | | |
| --- | --- | --- | --- | --- | --- | --- |
| ID | B | SE | Wald | Df | Sig | Exp(B) |
| F13A1 | .025 | .007 | 11.855 | 1 | .001 | 1.025 |
| FUCA1 | -.008 | .005 | 2.827 | 1 | .093 | .992 |
| FCGR2A | .034 | .017 | 3.882 | 1 | .049 | 1.035 |
| KCTD12 | -.016 | .008 | 4.079 | 1 | .043 | .984 |
| MFSD1 | -.080 | .028 | 8.248 | 1 | .004 | .923 |
| HLA-E | -.003 | .001 | 22.206 | 1 | .000 | .997 |
| SDCBP | .012 | .005 | 6.533 | 1 | .011 | 1.012 |
| MRC1 | -.071 | .015 | 23.793 | 1 | .000 | .932 |
| LCK | -.086 | .031 | 7.845 | 1 | .005 | .918 |
| PSME2 | .020 | .006 | 10.439 | 1 | .001 | 1.020 |
| VSIG4 | .016 | .007 | 5.435 | 1 | .020 | 1.017 |
| TAP2 | .215 | .038 | 32.022 | 1 | .000 | 1.240 |
| Df: Degree of freedom; Sig: Significance. | | | | | | |
